# CRISPR FISHer enables high-sensitivity imaging of nonrepetitive DNA in living cells through phase separation-mediated signal amplification

**DOI:** 10.1038/s41422-022-00712-z

**Published:** 2022-09-14

**Authors:** Xin-Yuan Lyu, Yuan Deng, Xiao-Yan Huang, Zhen-Zhen Li, Guo-Qing Fang, Dong Yang, Feng-Liu Wang, Wang Kang, En-Zhi Shen, Chun-Qing Song

**Affiliations:** 1 Fudan University, Shanghai, China; 2 Research Center for Industries of the Future, School of Life Sciences, Westlake University, Hangzhou, Zhejiang China; 3 Key Laboratory of Growth Regulation and Translational Research of Zhejiang Province, School of Life Sciences, Westlake University, Hangzhou, Zhejiang China; 4 Westlake Laboratory of Life Sciences and Biomedicine, Hangzhou, Zhejiang China; 5 Institute of Basic Medical Sciences, Westlake Institute for Advanced Study, Hangzhou, Zhejiang China

**Keywords:** Non-homologous-end joining, Biological techniques

## Abstract

The dynamic three-dimensional structures of chromatin and extrachromosomal DNA molecules regulate fundamental cellular processes and beyond. However, the visualization of specific DNA sequences in live cells, especially nonrepetitive sequences accounting for most of the genome, is still vastly challenging. Here, we introduce a robust CRISPR-mediated fluorescence in situ hybridization amplifier (CRISPR FISHer) system, which exploits engineered sgRNA and protein trimerization domain-mediated, phase separation-based exponential assembly of fluorescent proteins in the CRISPR-targeting locus, conferring enhancements in both local brightness and signal-to-background ratio and thus achieving single sgRNA-directed visualization of native nonrepetitive DNA loci in live cells. In one application, by labeling and tracking the broken ends of chromosomal fragments, CRISPR FISHer enables real-time visualization of the entire process of chromosome breakage, separation, and subsequent intra- or inter-chromosomal ends rejoining in a single live cell. Furthermore, CRISPR FISHer allows the movement of small extrachromosomal circular DNAs (eccDNAs) and invading DNAs to be recorded, revealing substantial differences in dynamic behaviors between chromosomal and extrachromosomal loci. With the potential to track any specified self or non-self DNA sequences, CRISPR FISHer dramatically broadens the scope of live-cell imaging in biological events and for biomedical diagnoses.

## Introduction

The genetic material, DNA, is dynamic and spatiotemporally organized to exert profound control over fundamental cellular processes and beyond.^1^ Genome instability or chromosomal structural variations could induce DNA damage and repair, sometimes generating extrachromosomal DNAs.^2^ In addition, some invaders, such as viruses, could infect the cell and deliver its genome into the nucleus, resulting in cellular dysfunction and disease development.^3^ Therefore, visualizing the spatial distribution and dynamics of nuclear DNA, including chromosomal and extrachromosomal DNA elements, is pivotal for understanding their biological functions.^4^ However, tracking any specified nuclear DNA in real time remains challenging in live cells.

Previous studies have achieved genomic imaging by integrating large arrays of artificial DNA sequences, such as LacO,^5^ which is dependent on tedious genome engineering for each locus of interest and may disturb the target loci and induce unpredicted side effects by these inserting exogenous sequences. Recently, given the specificity for Cas9 protein binding to target DNA guided by small sgRNA and a protospacer adjacent motif (PAM) in target sequences, CRISPR systems have been repurposed to image the dynamics and three-dimensional structure of endogenous genomic loci in live cells,^6^ overcoming the limitation of traditional fluorescence in situ hybridization (FISH), which requires fixed samples and denatured DNA.^7^ For example, fluorescent protein-fused deactivated Cas9 nuclease (dCas9)^6^ or dCas9 combined with sgRNA that recruits fluorescent protein-fused RNA binding proteins^8^ offers a good platform for live cell genomic imaging. Nevertheless, CRISPR-mediated live-cell imaging currently relies on multiple repetitive elements or an array of sgRNAs tiled along the target locus.^8–12^ In fact, few tandem DNA repeats for a specific locus exist in the human genome.^10,13^ The imaging strategy using sgRNAs tiling has trouble with achieving sufficient signal-to-background (S/B) ratio, making it challenging to visualize nonrepeated DNA sequences, thus precluding the widespread use of CRISPR imaging.^9^

Here, we report a DNA live-imaging system, CRISPR-mediated fluorescence in situ hybridization amplifier (CRISPR FISHer), based on phase separation-regulated local signal amplification, allowing for the robust visualization of native nonrepetitive sequences with a single sgRNA and expanding the application of CRISPR-mediated live-cell imaging.

## Results

### Design principle

**Fig. 1:**
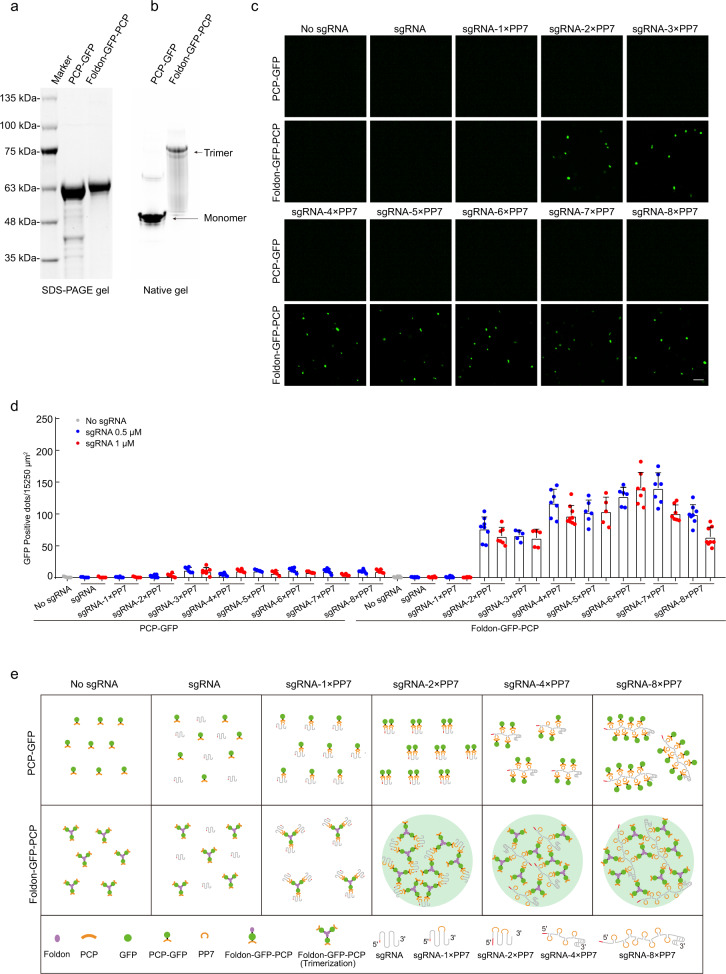
Trimeric foldon triggers the assembly of foldon-GFP-PCP protein and sgRNA with PP7 aptamers. **a**, **b** Purified proteins PCP-GFP and foldon-GFP-PCP separated by SDS-PAGE (**a**) and native PAGE (**b**). **c** Representative photomicrographs for PCP-GFP and foldon-GFP-PCP each incubating with a series of sgRNAs, including normal sgRNA or sgRNA with PP7 aptamers. In this assay, the concentrations of PCP-GFP, foldon-GFP-PCP and sgRNA were 1 μM, 1 μM, and 0.5 μM, respectively. The area for each field was 1695 μm^2^. **d** GFP dots per 15,250 μm^2^ after immediate incubation. The concentration of PCP-GFP and foldon-GFP-PCP each was 1 μM. **e** Proposed assembly mode for PCP-GFP or foldon-GFP-PCP with sgRNAs and engineered sgRNA with PP7 aptamers.

The design of CRISPR FISHer was based on the use of an engineered sgRNA scaffold to recruit RNA-binding motif-fused fluorescent proteins for programmable locus-specific imaging.^8^ In CRISPR FISHer, in addition to nuclease-deactivated dCas9, sgRNA with two PP7 RNA aptamers (sgRNA-2×PP7) was used to recruit a fusion protein consisting of the PP7 coat protein (PCP), green fluorescent protein (GFP), and a T4 fibritin trimeric motif foldon^14–16^ (termed foldon-GFP-PCP). As reported previously, purified foldon-GFP-PCP could form a soluble, stabilized trimer^15^ (Fig. 1a, b).

To determine whether the addition of trimeric foldon could induce the assembly of foldon-GFP-PCP protein and engineered sgRNA with PP7 aptamers, we incubated the purified foldon-GFP-PCP and PCP-GFP each with normal sgRNA or a series of engineered sgRNAs containing 1‒8×PP7 aptamers. Only in groups of foldon-GFP-PCP and sgRNAs with 2‒8×PP7, we observed a large number of small condensates, which displayed similar density among the groups (Fig. 1c, d). These findings indicate that trimeric foldon may trigger an exponential increase in the phase separation-based assembly of foldon-GFP-PCP and sgRNAs with 2‒8×PP7. The proposed models for assembly are shown in Fig. 1e. Given the ability of foldon to induce polymerization, CRISPR FISHer may increase local GFP accumulation at the sgRNA targeting locus in live cells.

### CRISPR FISHer enhances  S/B ratio at the targeting repetitive genomic loci through the assembly of foldon-GFP-PCP and engineered sgRNA

**Fig. 2:**
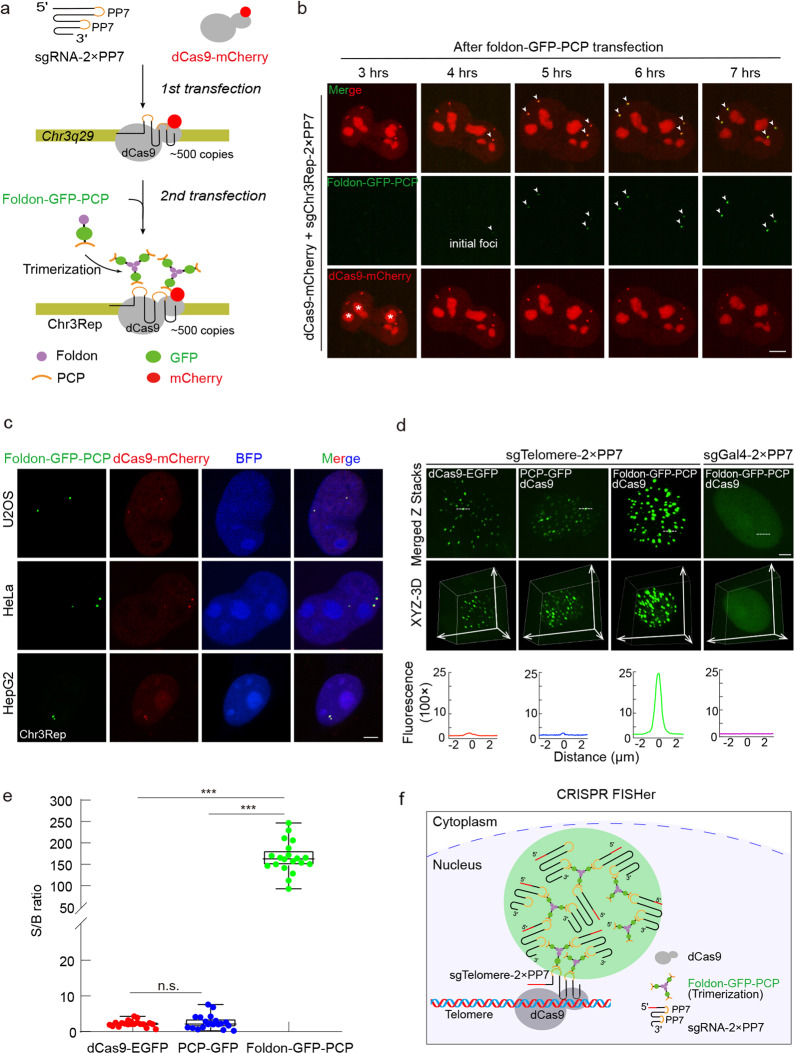
Foldon-GFP-PCP enables robust repetitive genomic loci tracking with enhanced S/B ratio by CRISPR FISHer. **a** Schematic diagram of CRISPR FISHer being recruited to the target site. Trimerized foldon-GFP-PCP was assembled at Chr3q29 locus (~500 copies, termed as Chr3Rep) targeted by dCas9-mCherry/sgChr3Rep-2×PP7 complex. **b** The enrichment of foldon-GFP-PCP at the Chr3Rep loci (arrows) labeled by dCas9-mCherry in live U2OS cell. Foldon-GFP-PCP foci started to occur ~4 h post transfection. The stars show nucleolar dCas9-mCherry accumulation. **c** Representative images showing colocalization of foldon-GFP-PCP (green) and dCas9-mCherry (red) on the Chr3Rep locus in U2OS, HeLa, and HepG2 cells. The plasmids expressing foldon-GFP-PCP, dCas9-mCherry, sgChr3Rep-2×PP7, and BFP were co-transfected. BFP was used as an indicator of the nuclei and sgRNA-2×PP7 expression. **d** Comparison of foldon-GFP-PCP, PCP-GFP, and dCas9-EGFP labeling of telomere loci in U2OS cells. sgGal4 is used as the negative control. The dotted lines (up) label area used to generate respective line scans (down). Middle, the spatial distribution of telomere loci. See Supplementary information, Video S1. **e** Comparison of S/B ratio of labeled telomere loci using foldon-GFP-PCP, PCP-GFP, and dCas9-EGFP. Data are presented as means ± SEM in **e** for dCas9-EGFP (2.273 ± 0.838, *n* = 20), PCP-GFP (2.619 ± 1.912, *n* = 20) and foldon-GFP-PCP (165.646 ± 36.038, *n* = 20). Two-tailed Student’s *t*-test was performed (ns nonsignificance; ***P < 0.001). **f** Schematic diagram of CRISPR FISHer at a telomere locus.

To investigate whether foldon-GFP-PCP could be recruited to and accumulate at the CRISPR target site in live cells, we first labeled a repeated genomic region at *Chr3q29* (~500 repeats, termed Chr3Rep) as a marker using dCas9-mCherry and Chr3Rep-targeting sgRNA-2×PP7 and subsequently nucleofected plasmid expressing foldon-GFP-PCP into human bone osteosarcoma U2OS cells (Fig. 2a). Imaging analysis showed that foldon-GFP-PCP foci appeared as early as 4 h after nucleofection, colocalized with Chr3Rep loci, and gradually became brighter and clearer, while the fluorescence intensity of dCas9-mCherry did not show notable enrichment (Fig. 2b). These results indicate that target DNA-bound dCas9/sgChr3Rep potentially recruited foldon-GFP-PCP to the targeting loci while enhancing the GFP signal at the target site and decreasing the nonspecific background. To exclude the effect of sequential delivery, we also co-transfected plasmids, including foldon-GFP-PCP, dCas9-mCherry and sgChr3Rep-2×PP7 into U2OS cells as well as HeLa and HepG2 cells for additional analysis of colocalization. As expected, foldon-GFP-PCP colocalized well with dCas9-mCherry 16 h after transfection (Fig. 2c). To further test the specificity of foldon-GFP-PCP localization induced by dCas9/sgRNA-2×PP7 at target sites, we applied another sgRNA targeting repetitive element at *Chr13q34* (~350 repeats, termed Chr13Rep) and verified the specific signal (Supplementary information, Fig. S1a).

Next, we labeled telomeres and compared CRISPR FISHer (dCas9/sgRNA-2×PP7/foldon-GFP-PCP) with two traditional CRISPR imaging systems (dCas9-EGFP/sgRNA-2×PP7 and dCas9/sgRNA-2×PP7/PCP-GFP) in U2OS cells. CRISPR FISHer targeting telomere loci displayed higher sensitivity (Fig. 2d; Supplementary information, Fig. S1b and Video S1), and its S/B ratio reached up to 246 (Fig. 2e), demonstrating that CRISPR FISHer significantly outperformed the two other CRISPR imaging systems in labeling repetitive genomic loci. CRISPR FISHer with sgGal4-2×PP7 (lacking a cognate target in the human genome), however, could not show specific foci in the nucleoplasm (Fig. 2d), suggesting that sgRNA targeting to DNA locus is important for foci formation. Consistent with previous reports that sgRNAs are highly unstable and easily degradable in living cells,^17,18^ the formation of dCas9:sgRNA:DNA ternary complex might be able to protect sgRNA from degradation by RNase.^12,19^ Therefore, we propose that the target DNA-bound dCas9:sgRNA might be stable and act as a “seed” to enable rapid aggregation of foldon-GFP-PCP and sgRNA-2×PP7 at the target DNA locus, maximize local GFP signals, and minimize the background noise in living cells (Fig. 2f).

### With a single sgRNA, CRISPR FISHer accomplishes the visualization of the endogenous nonrepetitive genomic region and exogenous HBV diagnosis in live cells

**Fig. 3:**
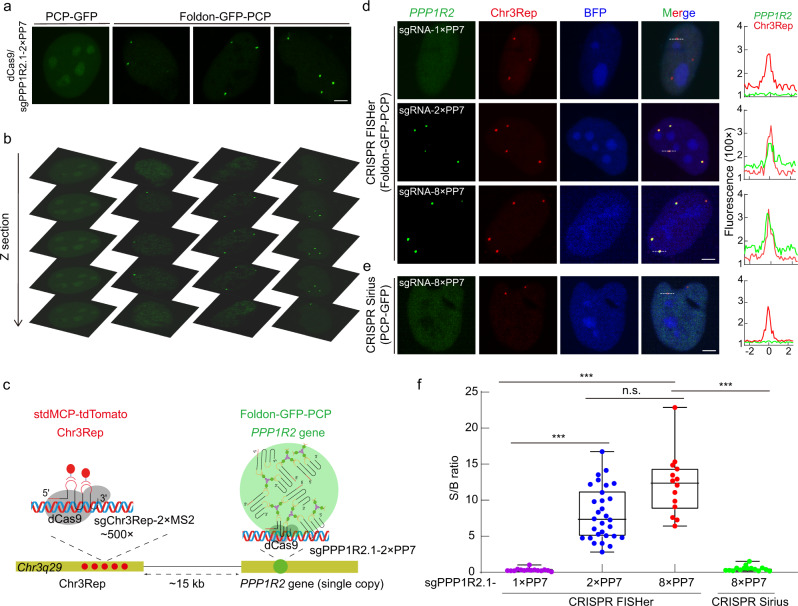
CRISPR FISHer achieves the visualization of the endogenous nonrepetitive genomic region. **a** Representative images for *PPP1R2* gene signal labeled by PCP-GFP or foldon-GFP-PCP. Scale bar, 5 µm. **b** Representative *PPP1R2* loci distribution in serial Z-sections for **a**. **c** Schematic diagram of dual-color CRISPR imaging for loci *PPP1R2* (GFP) and Chr3Rep (tdTomato). **d**, **e** Comparison of foldon-GFP-PCP and PCP-GFP labeling of single-copy gene *PPP1R2*. sgPPP1R2.1-1×PP7, sgPPP1R2.1-2×PP7 or sgPPP1R2.1-8×PP7 was used for targeting the *PPP1R2* gene (green); sgChr3Rep-2×MS2 was used for labeling Chr3Rep loci (red, internal control). The dotted lines (left) mark area for producing line scans (right). Scale bar, 5 µm. **f** Comparison of S/B ratio of labeled *PPP1R2* loci using CRISPR FISHer (foldon-GFP-PCP) and CRISPR Sirius (PCP-GFP). Two-tailed Student’s *t*-test was performed (ns nonsignificance; ***P < 0.001).

Nonrepetitive genomic regions account for ~65% of the human genome and include almost all protein-coding genes^13^ (Supplementary information, Fig. S2); given the power to enrich signals on the targeting sites, we tested the use of CRISPR FISHer to image the *PPP1R2* gene locus in nonrepeating genomic regions in live cells. The sgRNA targeting the *PPP1R2* gene (sgPPP1R2.1-2×PP7) was ~15 kb from Chr3Rep. We transfected the plasmids into dCas9-U2OS cells to express sgPPP1R2.1-2×PP7 and foldon-GFP-PCP or sgPPP1R2.1-2×PP7 and PCP-GFP. We observed 2–4 bright GFP puncta for foldon-GFP-PCP,^20^ but this was not observed in the control set with PCP-GFP (Fig. 3a, b), suggesting the capability of CRISPR FISHer to image *PPP1R2* gene loci and monitor the gene copy number in aneuploid U2OS cells.^10,20^ Additionally, in control data sets without dCas9 or with dCas9 and unrelated sgRNA (regular sgPPP1R2.1 or sgGal4-2×PP7), instead of forming specific foci, nuclear foldon-GFP-PCP tended to diffuse in the whole nucleus or accumulate in nuclei, presumably by nonspecific protein‒RNA interactions (Supplementary information, Fig. S3a). We also applied dCas9-mCherry in CRISPR FISHer and found the dCas9-mCherry was invisible at the nonrepetitive *PPP1R2* loci, suggesting that foldon-GFP-PCP and sgRNA-2×PP7 but not dCas9-mCherry might rapidly accumulate at the CRISPR- targeted loci (Supplementary information, Fig. S3b). Therefore, CRISPR FISHer is able to make native nonrepetitive regions visible in live cells.

To verify the specificity of the CRISPR FISHer-labeled nonrepetitive DNA region, we marked the *PPP1R2* gene using CRISPR FISHer and colabeled Chr3Rep using the 2×MS2 or 8×MS2 CRISPR system as the internal reference (Fig. 3c; Supplementary information, Fig. S4a). As expected, the loci targeted by CRISPR FISHer with sgRNA-2×PP7 or sgRNA-8×PP7 were highly colocalized with the internal reference signal in more than 90% of 300 GFP-positive U2OS cells (Fig. 3d; Supplementary information, Fig. S4b) as well as in most HeLa and HepG2 cells (Supplementary information, Fig. S5a), while CRISPR FISHer with sgRNA-1×PP7 did not show CRISPR targeting foci in U2OS cells (Fig. 3d). We also probed the sensitivity of CRISPR FISHer in the visualization of a nonrepeated sequence by comparing it with the sensitivity of another robust imaging system, CRISPR-Sirius (Fig. 3d–f; Supplementary information, Fig. S4c), which is capable of detecting as few as 22 repeats along genomic loci.^10^ The results showed that CRISPR-FISHer with sgRNA-2×PP7 or sgRNA-8×PP7, but not CRISPR-Sirius with sgRNA-8×PP7 or CRISPR-FISHer with sgRNA-1×PP7 (Fig. 3d–f; Supplementary information, Fig. S4b, c), enabled us to visualize the *PPP1R2* gene loci in live cells. These findings suggest that CRISPR FISHer is efficient and sensitive for single-copy gene imaging. Further experiments showed that foldon-PCP-GFP tended to accumulate at the target loci with a higher concentration of sgRNA (Supplementary information, Fig. S5b, c). In the following experiments, we mostly used sgRNA-2×PP7 for CRISPR FISHer because there was no significant difference in the S/B ratio between sgRNA-2×PP7 and sgRNA-8×PP7 (Fig. 3f).

**Fig. 4:**
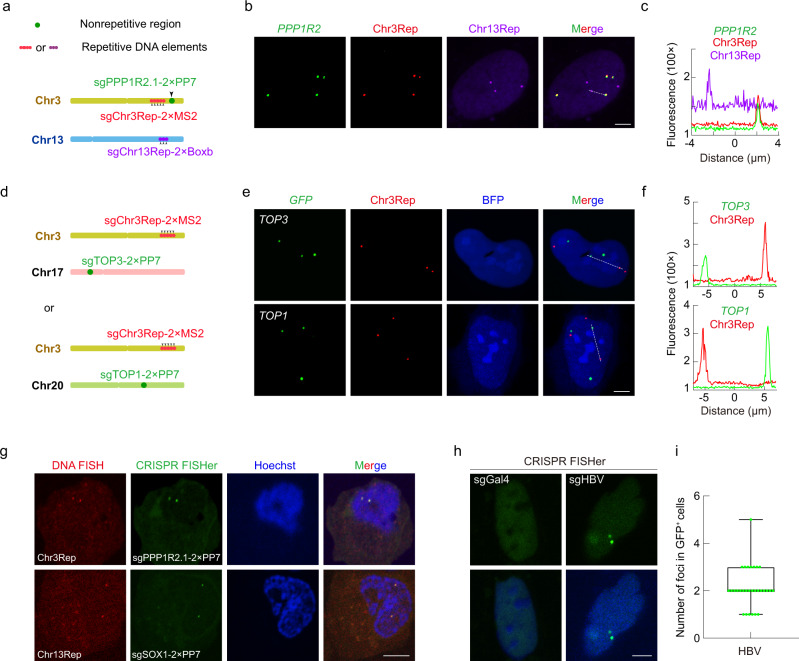
CRISPR FISHer allows for visualization of the endogenous nonrepetitive genomic region and exogenous HBV diagnosis in live cells. **a**, **b** Three-color CRISPR imaging of loci for *PPP1R2* gene (green), Chr3Rep (red), and Chr13Rep (purple) in U2OS cells. Schematic diagram of target loci on Chr3 and Chr13 (**a**). Representative in situ imaging for *PPP1R2* gene (green, foldon-GFP-PCP), Chr3Rep (red, stdMCP-tdTomato), and Chr13Rep (purple, N22-Halo) (**b**). Scale bar, 5 µm. Also see Supplementary information, Video S2. **c** Line trace showing the dotted line-marked area in **b**. **d**, **e** Visualization of single-copy gene *TOP3* or *TOP1* by CRISPR FISHer in U2OS cells. Schematic diagram of target loci on Chr3 and Chr17 or Chr20 (**d**). Representative images for *TOP3* or *TOP1* gene (green, foldon-GFP-PCP) and Chr3Rep (red, stdMCP-tdTomato, internal control) (**e**). Scale bar, 5 µm. **f** The line scan showing the dotted line-marked area in **e**. **g** DNA FISH and CRISPR FISHer foci pairs. Alexa Fluor 568 DNA FISH probe labeled Chr3Rep and Chr13Rep loci; CRISPR FISHer labeled *PPP1R2* and *SOX1* loci. Scale bar, 5 µm. **h**, **i** The visualization of HBV by CRISPR FISHer in live Hep3B cells. Representative HBV signal in Hep3B cells (**h**). sgGal4 is used as the negative control. Scale bar, 5 µm. The number of HBV loci in Hep3B cells (*n* = 30) (**i**).

Next, to further test the specificity of CRISPR FISHer in labeling nonrepetitive regions, we implemented five additional different strategies. First, we labeled another single-copy gene, *SOX1* (~250 kb from Chr13Rep in Chr13) (Supplementary information, Fig. S6a), and found that CRISPR FISHer-labeled *SOX1* gene foci colocalized well with Chr13Rep loci while CRISPR Sirius-labeled *SOX1* gene was invisible (Supplementary information, Fig. S6b, c). Second, we colabeled *PPP1R2* and two additional Chr3Rep and Chr13Rep loci with different fluorescent markers in the same cell (Fig. 4a). As a result, we detected robust *PPP1R2* gene loci marked by CRISPR FISHer that colocalized with Chr3Rep but not Chr13Rep (Fig. 4b, c; Supplementary information, Video S2). Third, we imaged the loci for Chr3Rep or Chr13Rep as well as single-copy genes, either *TOP3* on Chr17 or *TOP1* on Chr20 in U2OS cells (Fig. 4d; Supplementary information, Fig. S6d). The CRISPR FISHer signals for *TOP3* and *TOP1* did not merge with the signal for Chr3Rep (Fig. 4e, f), nor did they colocalize with Chr13Rep (Supplementary information, Fig. S6d, e). Fourth, we imaged *PPP1R2* and *SOX1* as well as Chr3Rep and Chr13Rep in a near-diploid human RPE cell line (Supplementary information, Fig. S1c, d). 86% and 93% of GFP-positive cells displayed two or four CRISPR FISHer GFP puncta for *PPP1R2* and *SOX1*, which colocalized well with Chr3Rep and Chr13Rep (93.5% and 94%, respectively) (Supplementary information, Fig. S5d–i). In addition, CRISPR FISHer was able to label the single copy gene *BAGE* and *TPTE* located in heterochromatin of RPE cells (Supplementary information, Fig. S5j). Finally, we performed standard DNA FISH to label Chr3Rep and Chr13Rep loci using Alexa Fluor 568 dye and confirmed that the CRISPR FISHer-marked *PPP1R2* and *SOX1* spots were colocalized with FISH probe-labeled repeated loci in U2OS cells (Fig. 4g). These results indicate that the CRISPR FISHer system enables precise visualization of nonrepetitive genomic regions within single live cells.

In addition, we extended the test of CRISPR FISHer to live Hep3B cells to visually detect hepatitis B virus (HBV), a major cause of life-threatening severe liver failure and liver cancer. Bright foci were observed with HBV-targeted sgRNA but not with sgGal4, agreeing with the previous report that Hep3B cells contain HBV integrants (Fig. 4h, i; Supplementary information, Fig. S6f, g).^21,22^

### CRISPR FISHer enables tracking of a consecutive process of DNA DSB-induced chromosomal dissociation and subsequent intra- and inter-chromosomal rejoining in live cells

**Fig. 5:**
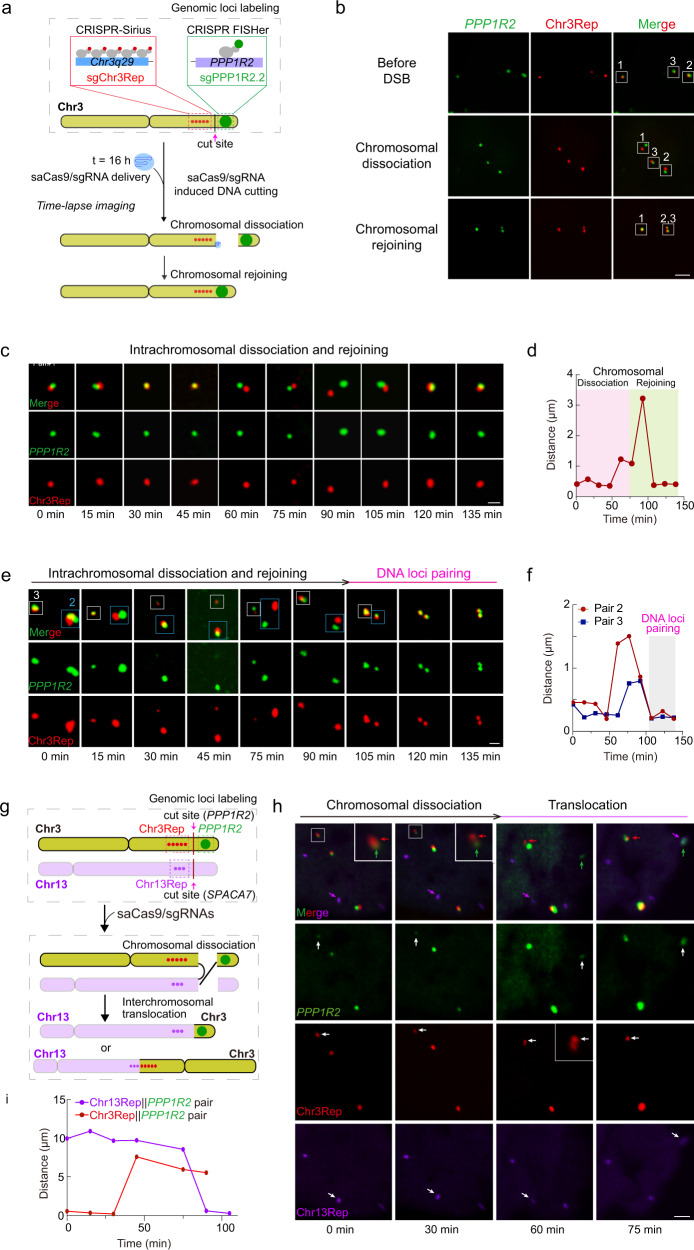
Simultaneous imaging of multiple CRISPR target loci tracks real-time dynamics of DNA DSB-induced chromosomal dissociation and subsequent intra- and interchromosomal rejoining in live cells. **a** Schematic diagram of intrachromosomal separation and rejoining through labeling both sides of the cutting site. Chr3Rep and *PPP1R2* gene on Chr3 were labeled by CRISPR Sirius and CRISPR FISHer in U2OS cells. SaCas9/sgRNA was delivered by nucleofection (16 h after delivering DNA loci labeling systems) for inducing DSB between the two labeled loci. **b** Representative fluorescent images of DSB-induced intrachromosomal dissociation and rejoining in a single cell. White boxes show DNA loci pairs. Scale bar, 5 µm. **c**, **d** Time-lapse imaging and corresponding distance of DNA loci of pair 1 in **b**. Scale bar, 1 µm. **e**, **f** Time-lapse imaging and corresponding distance of DNA loci of pairs 2 and 3 in **b**. After separation and association of Chr3 fragments, pairs 2 and 3 gradually gathered as shown in **e**. Scale bar, 1 µm. **g** Schematic diagram of DSB-induced interchromosomal translocation between Chr3 and Chr13. The labeling strategy is similar to Fig. 4a. saCas9/sgRNA was delivered to produce DNA cutting between the labeled loci on Chr3 and *SPACA7* gene on Chr13. **h** Representative images showing intrachromosomal dissociation and interchromosomal translocation between Chr3 and Chr13. Colored arrows indicate three DNA loci for tracking (green, *PPP1R2*; red, Chr3Rep; purple, Chr13Rep). The white box shows local enlargement. Time-lapse imaging started from 4 h post saCas9/sgRNA delivery. Scale bar, 1 µm. **i** Distance of DNA loci pairs shown in **h**. The red line shows the distance between Chr3Rep (red) and *PPP1R2* (green) paired foci; the purple line shows the distance between Chr13Rep (purple) and *PPP1R2* (green) paired foci.

The biomedical field has been revolutionized by CRISPR-mediated genome editing,^23–25^ which can generate DNA double-strand breaks (DSBs) that are repaired mainly by the nonhomologous end joining (NHEJ) pathway^26^ within the same chromosome. To monitor the dynamics of the intrachromosomal NHEJ process, different from the previous strategy of labeling one repetitive genomic loci and using a DNA damage sensor 53BP1 to monitor the local DSB processing,^12^ we labeled two ends of DSBs from the same chromosome in a single live cell. We first utilized CRISPR FISHer to label the *PPP1R2* gene with sgPPP1R2.2/foldon-GFP-PCP and CRISPR-Sirius to label Chr3Rep with sgChr3Rep/stdMCP-tdTomato in dCas9-U2OS cells; 16 h later, we nucleofected the saCas9/sgRNA plasmids targeting the *PPP1R2* gene to induce DSBs between these two tagged genomic loci on Chr3 in transfected cells (Fig. 5a). This sequential delivery strategy enabled us to track the dynamics of these two loci labeled with tdTomato and GFP before and after chromosomal breakage. In principle, after DSB-induced chromosomal dissociation, the paired red and green foci will experience the change in location patterns from colocalization to separation and consequent reunion. As expected, 4 h after active saCas9/sgRNA delivery, we captured the fission and fusion of three pairs of *PPP1R2* and Chr3Rep loci, likely indicating CRISPR-mediated cleavage of the target site at chromosome 3 followed by NHEJ-mediated repair within this time frame (Fig. 5b; Supplementary information, Fig. S7a). Overall, labeling two endogenous DSB ends enables the observation of the consecutive DSB-mediated NHEJ process in the same chromosome covering the initial chromosomal status before DSB as well as DSB-induced end separation and reconnection.

Furthermore, before the DSB, pair 1 was located a distance from pairs 2 and 3 and became closer after cutting (Fig. 5b), suggesting that the initial chromatin conformation was modified upon DNA damage to increase the spatial proximity between the DSB-containing chromosome domains. We observed the real-time movement of two loci labeled with GFP for *PPP1R2* gene and tdTomato for Chr3Rep (Fig. 5c, d), which maintained their subnuclear colocalization for at least 0.5 h and gradually separated with a distance of 3.24 µm, and finally associated together after separation for ~0.5 h, indicating the rapid dissociation and association of the two loci after CRISPR/saCas9-induced chromosomal cutting. We also observed similar dynamics for pairs 2 and 3 (Fig. 5e, f). Notably, after paired loci separation and association, the loci of pairs 2 and 3 became clustered, suggesting homologous pairing after NHEJ (Fig. 5e). We also noticed that the maximum distances between the two separated loci for pair 2 and pair 3 were 1.43 µm and 0.77 µm, in line with most observed NHEJ events (Supplementary information, Fig. S7d), supporting that after DSB, most of the paired foci were apart within 3 µm of each other before reunion as previously reported^27^(Fig. 5f), likely suggesting that after chromosome fracture, DNA damage- or repair-related proteins might facilitate two newly formed chromosomal ends to move closer for subsequent repair.

In addition to intrachromosomal repair, DSBs lead to interchromosomal recombination, such as translocations,^28^ which are tightly linked to cellular disorder and cancer. To visualize the dynamic process underlying chromosomal translocation, we applied the same labeling strategy used in Fig. 4a to mark the loci of the *PPP1R2* gene (GFP), Chr3Rep (tdTomato), and Chr13Rep (Halo), and we utilized the active saCas9/sgRNA editing system targeting both Chr3 and Chr13 to induce DSBs. One break site on Chr3 was located between the sgPPP1R2.2- and sgChr3Rep-targeted loci; the other was on the *SPACA7* gene, 82 kb from the Chr13Rep region (Fig. 5g). After nucleofection of saCas9/sgRNA, we followed the process of chromosomal breakage and translocation. Initially, the sgPPP1R2.2- and sgChr3Rep-targeted loci on Chr3 overlapped (GFP/tdTomato) for over 6 h, and they then segregated as far apart as 7.59 µm; finally, CRISPR FISHer-labeled *PPP1R2* gene loci colocalized with sgChr13Rep-targeted loci on Chr13 (GFP/Halo) (Fig. 5h, i), likely suggesting that breakage-induced small chromosomal segments moved far away, resulting in the failure of intrachromosomal repair, and the eventual repair through inter-chromosomal end joining. We also observed another translocation event between two large fragments of Chr3 and Chr13, consistent with the previous report^12^ (Supplementary information, Fig. S9a–c). Moreover, we observed the inter- and intra-chromosomal NHEJ repairs within one single cell (Fig. 5h), indicating that labeling three native loci allow multiple possible NHEJ processes to be tracked on the same or different chromosomes after DSB and suggesting that the distance between DSB ends may determine the selection of inter- or intra-chromosomal repair. Finally, we verified these chromosomal translocation events by targeted sequencing (Supplementary information, Figs. S8, S9).

### CRISPR FISHer detects extrachromosomal circular DNA elements and visualizes their dynamics in live cells

**Fig. 6:**
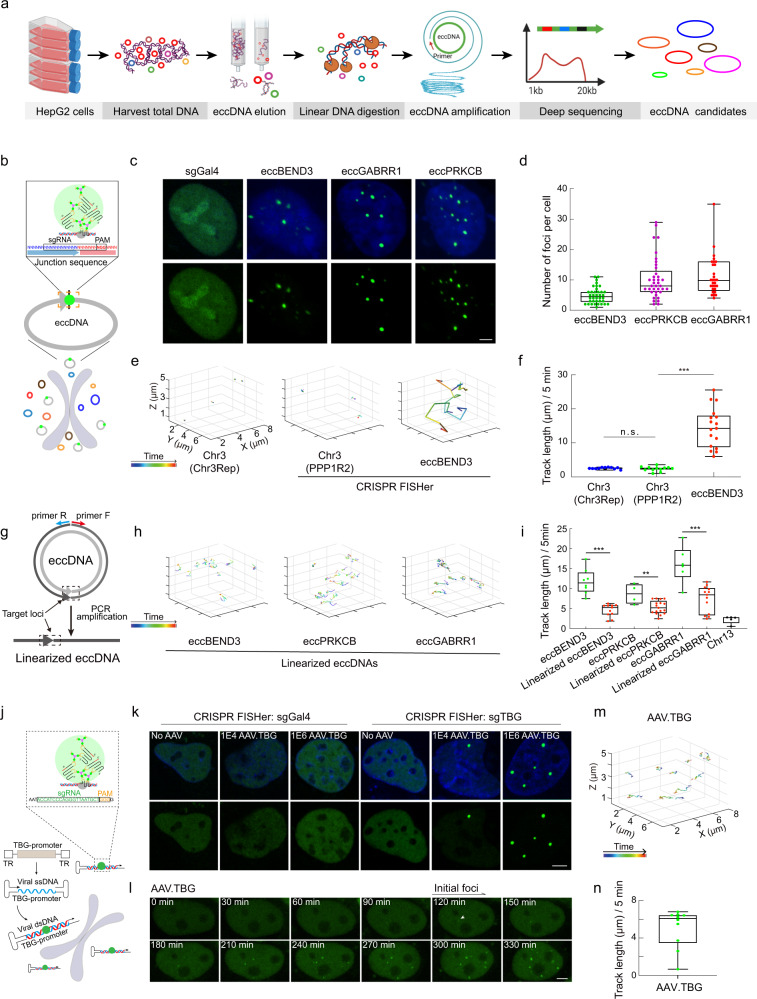
CRISPR FISHer detection and real-time visualization of native extrachromosomal eccDNAs and invading AAV DNA in live cells. **a** Schematic diagram of the isolation, enrichment, amplification, and deep sequencing of eccDNAs from HepG2 cells. **b** The schematic strategy of the eccDNA labeling with CRISPR FISHer. sgRNA target sites are located at junction regions of eccDNAs. **c**, **d** Representative images showing labeled eccDNAs by CRISPR FISHer in HepG2 cells (**c**) and the number of eccDNA foci (**d**). **e** Representative XYZ-t trajectories for eccBEND3 and Chr3 loci during a 5-min period. See Supplementary information, Videos S3‒S5 for dynamics. **f** Comparison of tracking length of eccBEND3 and Chr3 in HepG2 cells. Two-tailed Student’s *t*-test was performed (ns nonsignificance, ***P < 0.001). **g** The schematic diagram of the linearized eccDNA amplified from eccDNA by PCR. Dotted boxes indicate the CRISPR FISHer targeting locus as well as junction regions of eccDNA. **h** Representative XYZ-t trajectories for linearized eccDNAs during a 5-min period. See Supplementary information, Videos S9‒S11 for dynamics. **i** Tracking length of circular and linearized eccDNAs as well as Chr13. Two-tailed Student’s *t*-test was performed (ns nonsignificance, ***P < 0.001). **j** Schematic diagram showing the labeling strategy of adeno-associated virus (AAV) with CRISPR FISHer. **k**, **l** Representative images showing nuclear ds AAV DNA loci labeled by CRISPR FISHer in U2OS cells. The appearance and increasing formation of ds AAV DNA foci over time were shown in a single live cell (**l**). The sgRNA targeting mouse TBG carried by AAV was used. 1E4 or 1E6 particles of AAV.TBG was used for detection. **m**, **n** Representative trajectory (**m**) and tracking length (**n**) for ds AAV DNA loci in U2OS cells. See Supplementary information, Video S12 for dynamics. sgGal4 was used as the control sgRNA in **c** and **k**. Scale bars in **c**, **k** and **l**, 5 µm.

Beyond the enormous genome DNA molecules, cells contain other DNA types. For example, extrachromosomal circular DNA elements (eccDNAs), firstly reported in 1964, exist in most tissues and cell lines and range in size from hundred to million bases.^28^ The extrachromosomal DNAs (called ecDNA), ranging from 50 kb to 5 Mb, could be detected by DNA FISH. However, it is challenging to visualize eccDNAs by DAPI or DNA FISH due to their small size. Recent studies have highlighted the functions of eccDNAs in gene regulation, the immune response, and cellular communication.^29–31^ We applied CRISPR FISHer to study eccDNAs in live cells. First, we isolated and sequenced the eccDNAs from HepG2 cells (Fig. 6a). Among them, the sequences of eccBEND3, eccGABRR1, and eccPRKCB were independently verified using three rounds of PCR, TA cloning, and Sanger sequencing (Supplementary information, Fig. S10a–d). The eccDNA junction sequences were selected as CRISPR FISHer targeted sites (Supplementary information, Fig. S10b) because they are unique and not present in the human genome, enabling specific targeting by CRISPR FISHer (Fig. 6b). We observed the spatial distribution of CRISPR FISHer-targeted loci in HepG2 cells (Fig. 6c) and calculated the number for each eccDNA (Fig. 6d), indicating the feasibility of CRISPR FISHer in labeling and tracking eccDNA.

Next, we followed the spatiotemporal dynamics of individual loci for eccBEND3 as well as sgPPP1R2.2-targeted loci for Chr3 (Fig. 6e; Supplementary information, Videos S3–S5) and found that the average traveling distance and space of the eccBEND3 loci exceeded those of Chr3 within the same period (Fig. 6e, f), revealing that eccDNA is highly dynamic with a longer trajectory and faster movement. We further confirmed these differences in dynamics by tracking the real-time movement of another two eccDNAs and Chr13 (Supplementary information, Fig. S10e and Videos S6‒S8). Moreover, we PCR-amplified the linear version of eccDNA, transfected the linearized eccDNA with the same length and sequence as the detected eccDNA into a U2OS cell line that is devoid of the endogenous version of this eccDNA, and analyzed the behavior of the linear version using the CRISPR FISHer by targeting the junction sequence of eccDNA, and tracked its cellular dynamics (Fig. 6g, h). We found that the native circular eccDNAs moved faster than their linearized ones, indicating that the circularity of eccDNA is critical for its fast movement (Fig. 6i; Supplementary information, Videos S3, S7‒S11). This peculiar dynamic feature of eccDNA suggests that it might serve as a mobile signal transduction molecule in a circularity-dependent manner, in line with the previous report that the circularity of eccDNA, but not its linearity or sequence, is required for eccDNA to act as an innate immunostimulant.^30^

### CRISPR FISHer detects invading AAV DNA and tracks its dynamics in live cells

Finally, we extended the usage of CRISPR FISHer to imaging invading DNA in real time. Adeno-associated virus (AAV) is an attractive vector in human gene therapy because it is a nonpathogenic parvovirus.^32^ Double-strand (ds) AAV DNA, generated from replication of its single-strand DNA, enables CRISPR FISHer targeting (Fig. 6j). We first delivered CRISPR FISHer targeting the rAAV genome TBG into U2OS cells by nucleofection. Twelve hours later, the CRISPR FISHer GFP signal diffused through the nucleus, and AAV particles were added for infection. Specific GFP foci were observed after cell treatment with both AAV and the sgTBG plasmid but not detected in the absence of AAV infection or sgGal4 plasmid transfection (Fig. 6k, l), indicating that CRISPR FISHer enables ds AAV DNA labeling in a live cell. Notably, we observed the emergence of ds AAV DNA after AAV infection (Fig. 6l), suggesting that CRISPR FISHer helps evaluate the effective number of AAV DNA molecules in live cells. We visualized the spatiotemporal dynamics of ds AAV DNA loci and discovered that individual loci showed high mobility, comparable to that of eccDNA, but traveled within a small space (Fig. 6m, n; Supplementary information, Video S12), suggesting that AAV dsDNA was trapped in a confined area, which may be related to and benefit its transcription.

## Discussion

Here, we report a versatile system called CRISPR FISHer, which accomplishes live-cell imaging of nonrepeated DNA sequences from endogenous or exogenous DNAs (Supplementary information, Fig. S11). The principle of CRISPR FISHer is based on the trimeric protein motif and engineered sgRNA-triggered, phase separation-mediated exponential GFP accumulation at the target locus, which profoundly improves the target signal while reducing background noise. The traditional CRISPR labeling strategies could detect the repetitive telomere loci (the medium S/B ratio is less than 3) (Fig. 2e) but not nonrepetitive loci, whereas CRISPR FISHer with a single sgRNA is able to indicate the native nonrepetitive *PPP1R2* gene locus (S/B ratio reaching up to 22) (Fig. 3f). Therefore, the significant improvement of the S/B ratio enables CRISPR FISHer as a sensitive and easy-to-use tool to detect and track target DNA loci of interest using the standard confocal microscopy systems.

We investigated the potential of this system for monitoring fundamental nuclear processes by tracking the dynamics of labeled DSB-induced chromosomal fragments, eccDNAs, and invading viral DNA. The spatiotemporal dynamics of dissociated chromosomal fragments indicate that the distance between DSB-induced dual ends correlates with the selection of inter- or intra-chromosomal NHEJ repair. Furthermore, most broken DSB chromosomal ends are trapped in less than 3 µm,^27^ suggesting that DNA damage or repair-related proteins such as ku70/ku80 and 53BP1 may bind to these broken ends and restrict their movement. The live imaging for visualizing the dynamics of DSB-mediated DNA broken ends and NHEJ-related proteins will help further understand the stepwise process of NHEJ. Observing these DNA repair processes will further our understanding of numerous human diseases, including cancer and aging, caused by genome instability. In addition to genomic DNA, CRISPR FISHer enables tracking of the spatiotemporal dynamics of extrachromosomal DNAs such as eccDNA. These results reveal that the nature of non-integrated extrachromosomal DNAs is distinct from that of chromosomal DNA. Considering that CRISPR FISHer may interfere with the movement of the detected target, further studies are required to optimize the system.

This study sets the foundation for further understanding multiple biological events in live cells by combining orthogonal Cas9s, sequencing such as Hi-C, and real-time imaging. The CRISPR FISHer here achieved imaging of single nonrepetitive sequences in living cells, and it will be worthwhile to achieve multiple DNA sequence labelings by exploring more imaging systems using different RNA ligand-binding systems such as boxB-N22p coupling with other phase separation regions. Together with RNA and protein imaging tools, multiple DNA sequence labelings may facilitate the study of 4D nucleome, including spatiotemporal organization of the human genome, genomic interactions among different elements (e.g., enhancer and promoter), and real-time transcription of genes of interest in live cells. Furthermore, CRISPR FISHer allows the study of the replication, integration into the host genome, and the response of the innate immune system of invading DNAs in a single live cell, which may aid in biomedical diagnosis and clinical treatment.

## Materials and Methods

### Plasmid construction

Plasmids expressing stdMCP-tdTomato (Addgene 164044) or PCP-GFP (Addgene 121938) are gifts from Baohui Chen and Thoru Pederson. SpCas9-related sgRNAs used in this study were constructed by modifying pPUR-mU6-sgRNA-Sirius-8×PP7 (Addgene 121943) or pPUR-mU6-sgRNA-Sirius-8×MS2 (Addgene 121942). The fragments of sgRNA-2×PP7 (Addgene 75390), sgRNA-2×MS2 (Addgene 75389), and sgRNA-2×boxB (Addgene 75391) were subcloned into the backbone vector by NEB Gibson assembly kit (E2611L). The plasmid expressing foldon-GFP-PCP was constructed by inserting T4 fibritin trimeric motif foldon^14^ at the N- or C-terminus of PCP-GFP or GFP-PCP by Gibson assembly (NEB, E2611L). We compared various versions and selected the version of foldon-GFP-PCP in this study. The plasmid for N22-Halo was constructed by replacing the MCP domain of vector MCP-Halo (Addgene 121937) with the N22 domain.^8^ The all-in-one saCas9/sgRNA plasmid contained saCas9 and sgRNAs targeting Chr3 and Chr13. For plasmids expressing sgRNA, hPGK (promoter)-BFP-NLS was used as a reporter for sgRNA expression and an indicator for the nucleus. All sgRNAs used are listed in Supplementary information, Fig. S13. sgRNA binding protein sequences are in Supplementary information, Fig. S14.

### Protein purification

The DNA fragments of foldon-GFP-PCP and PCP-GFP were amplified by PCR and cloned into the pET-30a (+) bacterial expression vector containing a C-terminal His tag (Genscript). The protein was expressed in BL21(DE3) cells (Vazyme, C502) cultured in LB medium over 18 h of induction with 1 mM isopropyl-D-thiogalactopyranoside (IPTG) at an OD_600_ of 0.8 at 18 °C. Cells were harvested and frozen in liquid nitrogen for storage at −80 °C. All procedures were performed at 4 °C. After thawing, the cells were resuspended in 20 mM Tris-HCl, pH 7.5, 1.0 M KCl, 30 mM imidazole, 1 mM TCEP, 1 mM PMSF, and proteinase inhibitor (Roche, 05892791001), and lysed with an ultrasonic cell disruptor (Fisherbrand, FB705). After centrifugation (20,000× *g*, 1 h, 4 °C), the supernatant was loaded onto a Ni Sepharose 6 Fast Flow affinity column (Cytiva, 17531803) equilibrated in 20 mM Tris, pH 7.5, 1.0 M KCl, and 30 mM imidazole. Nonspecifically bound proteins were washed away with the same buffer containing 50 mM imidazole. Then, the target proteins were eluted with 1.0 M imidazole in the same buffer. The eluate was concentrated in a Centricon-50 (Millipore, UFC901096) and further purified by chromatography on a Superdex^TM^ 200 increase 10/300 GL column (Cytiva, 10280712) equilibrated with 20 mM HEPES, pH 7.5, 500 mM KCl, and 0.5 mM TCEP.

### In vitro phase-separation assay

To study the phase separation, the proteins of interest (PCP-GFP and Foldon-GFP-PCP) were suspended in 500 mM KCI and 20 mM HEPES buffer, pH 7.5. The RNA samples were synthesized by HiScribe^TM^ T7 Quick High Yield RNA Synthesis Kit (NEB, E2040S) and purified by RNA Clean & Concentrator^TM^-5 Kit (ZYMO RESEARCH, R1015). A total 10 μL of the mixture was prepared under 150 mM final salt concentration, and 2 μL was transferred to a sandwiched chamber created by a cover glass. All images were captured at room temperature (RT) within 5 min for the mixed samples. Samples were observed by Dragonfly 200 (Andor, Dragonfly 200).

### SDS-PAGE (denaturing PAGE) and native PAGE for purified proteins

The purified foldon-GFP-PCP and PCP-GFP proteins were diluted to 1 μg/µL. Native PAGE was carried out as previously described with modification.^15^ Twenty micrograms of protein were loaded onto precast polyacrylamide gels (LABLEAD, P42011) after being mixed with native loading (Solarbio Life Science, P1017). The gels were photographed by ChemiDocTM Imaging System (BIO-RAD, 12003153) with a 488-nm laser after electrophoresis. For SDS-PAGE, precast polyacrylamide gels (GenScript, M41212C) were loaded with 20 μg of protein that was previously mixed with 5× loading buffer (with 10% SDS and 10% β-mercaptoethanol) and then heated at 100 °C for 10 min. The gels were stained with Coomassie Blue for 10 min after electrophoresis. Before imaging, the gels were decolored with 10% acetic acid and 30% ethanol for 5 h.

### Cell culture and transfection

Human U2OS, HeLa, HepG2, Hep3B, hTERT RPE-1(RPE), and 293 T cells from ATCC were cultured at 37 °C in high-glucose DMEM (HyClone, SH30243.01) supplemented with 10% (vol/vol) FBS (GIBICO, 10099141 C) and 1% penicillin-streptomycin (Hyclone, SV30010). Transfection was carried out with Lipofectamine^®2000^ (Thermo Fisher, 11668019) and Lipofectamine^®3000^ (Thermo Fisher, L3000015) according to the manufacturer’s instructions. Briefly, after 20 min of incubation at RT, the DNA-Lipofectamine complex was added to 20-mm glass-bottom cell culture dishes (NEST, 801001) with 70%‒90% cell coverage. For nucleofection, 4D-nucleofector (LONZA, AAF-1002B, AAF-1002X, AAF-1002Y, and AAF-1002L) transfection was carried out according to the manufacturer’s instructions. Briefly, 150 ng of foldon-GFP-PCP, 100 ng of dCas9, and 100 ng of sgRNA plasmid were transfected into 2 × 10^5^ cells under the CM-104 program, and the transfected cells were incubated in a CO_2_ incubator (Thermo Scientific, 4111FO). For target loci imaging, the concentration of foldon-GFP-PCP protein in the nucleoplasm was suggested to be at 2.97~4.67 μM.

To image cells that did not express the BFP nuclear indicator, Hoechst 33342 (Thermo Fisher, H3570) was used at a final concentration of 5 μg/mL to stain the nuclei. To image the transfected cells showing a Halo signal, Janelia Fluor^®^ 646 HaloTag^®^ Ligand (Promega, GA1121) was added 15 min before imaging at a final concentration of 5 nM.

### Generation of the dCas9-U2OS foldon-GFP-PCP/stdMCP-tdTomato stable cell line

dCas9, foldon-GFP-PCP, and stdMCP-tdTomato lentivirus were prepared as previously described.^33,34^ U2OS cells were infected with dCas9 lentivirus and selected with blasticidin to obtain a dCas9 stable cell line. The dCas9-U2OS cells were sequentially infected with foldon-GFP-PCP lentivirus and stdMCP-tdTomato lentivirus and selected by fluorescence-activated cell sorting (FACS) (Sony, MA900) to generate the dCas9-U2OS/foldon-GFP-PCP and dCas9-U2OS/stdMCP-tdTomato cell lines. The stable cell line expressing foldon-GFP-PCP is suggested to be used freshly.

### Fluorescence microscopy

Generally, imaging was performed at 12‒36 h post-transfection. Images were acquired with the Dragonfly 200 (Andor, Dragonfly 200) using a Sona camera (Andor, SONA-4BV6U) and a Plan APO λ 100× / 1.45 Oil ∞ / 0.17 WD 0.13 objective (Nikon, CFI Plan Apochromat Lambda 60XC) mounted on a Leica DMi8inverted 20 microscopes. A 200-mW solid-state 405-nm laser and 445/46-nm BP emission filter were applied for BFP. GFP images were acquired with a 150-mW solid-state 488-nm laser and 521/38-nm BP emission filter. Red (tdTomato) images were acquired with a 150-mW solid-state 561-nm laser and 594/43-nm BP emission filter. Purple (Halo) images were acquired with a 150-mW solid-state 637-nm laser and 698/77-nm BP emission filter. Unless specifically indicated, Z-stack images were captured at a 0.2 µm step size to include all foci of each nucleus examined. For XYZ-t 4D imaging, cells were incubated in a Bold Line Cage Incubator (Okolab, H201-PRIOR-H117) equipped with a Dragonfly 200 at 37 °C and 5% CO_2_.

### Imaging processing and analysis

Imaris9.3.1 (Bitplane) was used to measure spot distances. Z-series acquired at a 0.2 µm step size were used. For each channel, spots were segmented based on the maximum intensity in the 3D volume of the nucleus. Measurement points were set to intersect 10 with the center of the spot object. Then, with line mode set as pairs, distances between locus pairs in the 3D volume were measured from a spot in one channel to the closest spot in the other channel. Track lengths were measured according to the manufacturer’s instructions of the Sport/Track module in imaris. For S/B ratios calculation, we use the mathematical formula: (signal intensity of the target locus − signal intensity of the nuclear background) / (signal intensity of the nuclear background − camera baseline).

### Visualization of chromosomal fragment dynamics and subsequent intra- and inter-chromosomal repair

To monitor the dynamics of chromosomal fragments and NHEJ-mediated intrachromosomal repair, plasmids expressing deactivated SpCas9-based CRISPR FISHer/GFP and CRISPR-Sirius/tdTomato were transfected into U2OS cells to label the *PPP1R2* gene and Chr3Rep, respectively. 16 h later, 100 ng of the saCas9/sgRNA system targeting the *PPP1R2* gene were nucleofected to induce chromosome cutting between the labeled loci on Chr3. The GFP and tdTomato double-positive cells were chosen for imaging 4‒6 h after nucleofection every 15 min. To visualize interchromosomal translocation events, the dCas9-based orthogonal sgRNA CRISPR imaging was applied to label the loci of the *PPP1R2* gene using sgRNA-2×PP7 and foldon-GFP-PCP, Chr3Rep using sgRNA-8×MS2 and stdMCP-tdTomato, and Chr13Rep using sgRNA-2×Boxb and N22-Halo. Then, 16 h later, 200 ng of active saCas9/sgRNA editing systems targeting both Chr3 and Chr13 were delivered. The cutting site on Chr3 was between the sgPPP1R2.2 (GFP) and sgChr3Rep (tdTomato) targeting loci, and the one on Chr13 was in the *SPACA7* gene, 82 kb from the Chr13Rep (Halo) region. Generally, 6 h after nucleofection, the GFP, tdTomato, and Halo triple-positive cells were chosen for imaging every 15 min and tracked for at least 10 h. The sgRNAs used here are listed in Supplementary information, Fig. S13.

### DNA FISH

DNA FISH was modified as described previously.^35^ After attaching to the cover-glass surface, cells were fixed with 4% paraformaldehyde solution at RT for 10 s and washed with PBS buffer three times (5 min/each). Then cells were permeabilized by methanol for 1 min followed by PBS buffer wash (5 min/each) and heated on a hot plate at 80 °C for 10 min in 80% formamide (Sangon Biotech, A600212). Next, cells were incubated for 1 min in a hybridization solution of 200 nM oligo probes (Sangon Biotech (Shanghai)) in the presence of 50% formamide and 2× SSC at 75 °C for 2 min. Finally, cells were washed by PBS buffer three times at RT (7 min/each).

Alexa Fluor 568 probe-based DNA FISH sequence to image human genomic loci are shown as follows:

Chr3: 5′-CCACTGTGATATCATACAGAGG-3′

Chr13: 5′-GGTAAGCATGGACCATTCCTTC-3′.

### PCR, cloning, and sequencing of translocation segments

For the visible interchromosomal translocation between Chr3 and Chr13, PCR primers were designed upstream or downstream of the cutting site of the saCas9 system on Chr3 and Chr13. The genome was harvested after imaging using QuickExtract™ DNA Extraction Solution (Lucigen, QE09050) according to the manufacturer’s instructions. The specific primer pairs were chosen for the connections between the GFP/Halo signals or tdTomato/Halo signals. The details are shown in Supplementary information, Figs. S8, S9. The PCR was performed with the Taq enzyme, and the purified PCR products were inserted into the TA vector for Sanger sequencing. Primers used here are listed in Supplementary information, Fig. S12.

### Enrichment, amplification, sequencing, and analysis of eccDNA

To extract eccDNA, 1 × 10^7^ HepG2 cells were used to harvest the whole-genome DNA according to the manufacturer’s instructions (Tiangen, DP304-03). Plasmid-Safe ATP-dependent DNase (Lucigen, E3110K) was used to digest the remaining linear DNA after eluting column-bound DNA. The DNase was heat-inactivated for 30 min at 70 °C. Then, the eccDNA was amplified by ϕ29 polymerase (Qiagen, P7020-HC-L) at 30 °C for 16 h according to the manufacturer’s instructions. The linear eccDNA product was sent for next-generation sequencing (Diatre Biotechnolog). Raw reads were trimmed by the Trim Galore software (version 0.5.0) (https://github.com/FelixKrueger/TrimGalore). The cleaned reads were aligned to the UCSC human hg19 genome using BWA (version 0.7.17).^36^ Next, the software samtools (version 1.8) was used to convert the aligned sam files into bam files.^37^ Then Circle-Map (version 1.1.3) was used to identify the circular DNA, and the finally generated BED file contained the genomic coordinate information of circular DNA. According to the circle score, the circles with high values were sorted out.^38^ Finally, the annotation of genome position was performed using the software bedtools (version 2.17.0).^39^

### Verification and visualization of eccDNA

Based on the deep sequencing results, three rounds of PCR combined with Sanger sequencing were performed to verify the sequences of eccDNAs. The junction sequences at the cyclization site of the verified eccDNAs were targeted by CRISPR FISHer for imaging.

To visualize the trajectories of exogenous linearized eccDNAs, the linearized eccDNAs were amplified and purified from HepG2 cells, and then transfected into U2OS cells. Briefly, 150 ng of foldon-GFP-PCP, 100 ng of dCas9, 100 ng of sgRNA plasmids, and 200 ng of linear eccDNA were transfected into 2 × 10^5^ U2OS cells using CM-104 program through nucleofection, and the transfected cells were incubated in a CO_2_ incubator until imaging.

### Detection and visualization of HBV

Three pairs of primers were designed based on the HBV genome sequence from NCBI. The HBV sequence was amplified using the enzyme Taq. As a result, only the S gene of HBV was amplified in Hep3B cells, and the PCR products were cloned into a TA vector for sequencing. The sgRNA for imaging was designed according to the sequences of the verified HBV S gene. The primers and sgRNAs are shown in Supplementary information, Figs. S12, S13.

### Detection and visualization of AAV

The U2OS cells were nucleofected with 150 ng of foldon-GFP-PCP, 100 ng of sgTBG-2×PP7 targeting the rAAV genome, and 150 ng of dCas9 plasmids through a 4D-nucleofector. Then, 12 h later, the cells were infected with AAV (WZ Biosciences Inc.), and diluted in a complete culture medium. The imaging detection started 12‒24 h post-infection.

### Statistics of chromosome-specific repeats in the human genome

We analyzed the resulting chromosome-specific loci of different copies in the human genome (assembly GRCh37/hg19). All results were obtained from the online tool CRISPRbar (http://genome.ucf.edu/CRISPRbar/), including sequence copy number information on all chromosomes.^8^ In addition, the R package “ggbio” (version 1.42.0) was implemented to plot the chromosome copy number distribution.^40^ The following chromosome-specific repeats were used in this study: Chr3q29: 195199022‒195233876; Chr13q34: 112930173‒112968847. The sgRNA sequences are listed in Supplementary information, Fig. S13.

### Statistical analysis

All statistical data are shown as means ± SEM of at least three replicates using GraphPad Prism (San Diego, CA, USA, version9.3.1). Two-tailed Student’s *t*-test was used to determine the *P* value between two groups. For all figures, *, ** and *** indicate *P* < 0.05, *P* < 0.01 and *P* < 0.001, respectively, and ns indicates no significance.

## Supplementary information

Fig. S1

Fig. S2

Fig. S3

Fig. S4

Fig. S5

Fig. S6

Fig. S7

Fig. S8

Fig. S9

Fig. S10

Fig. S11

Fig. S12

Fig. S13

Fig. S14

Supplementary Video S1

Supplementary Video S2

Supplementary Video S3

Supplementary video S4

Supplementary Video S5

Supplementary Video S6

Supplementary Video S7

Supplementary Video S8

Supplementary Video S9

Supplementary Video S10

Supplementary Video S11

Supplementary Video S12

Supplementary video legends

## Data Availability

All data are available in the main text or Supplementary information from corresponding authors upon request. In addition, plasmids will be available on Addgene.
