# Correction: CRISPR FISHer enables high-sensitivity imaging of nonrepetitive DNA in living cells through phase separation-mediated signal amplification

**DOI:** 10.1038/s41422-022-00742-7

**Published:** 2022-11-14

**Authors:** Xin-Yuan Lyu, Yuan Deng, Xiao-Yan Huang, Zhen-Zhen Li, Guo-Qing Fang, Dong Yang, Feng-Liu Wang, Wang Kang, En-Zhi Shen, Chun-Qing Song

**Affiliations:** 1 Research Center for Industries of the Future, School of Life Sciences, Westlake University, Hangzhou, Zhejiang China; 2 Key Laboratory of Growth Regulation and Translational Research of Zhejiang Province, School of Life Sciences, Westlake University, Hangzhou, Zhejiang China; 3 Westlake Laboratory of Life Sciences and Biomedicine, Hangzhou, Zhejiang China; 4 Institute of Basic Medical Sciences, Westlake Institute for Advanced Study, Hangzhou, Zhejiang China

**Keywords:** Non-homologous-end joining, Biological techniques

Correction to: *Cell Research* 10.1038/s41422-022-00712-z, published online 14 September 2022

The article “CRISPR FISHer enables high-sensitivity imaging of nonrepetitive DNA in living cells through phase separation-mediated signal amplification”, written by Xin-Yuan Lyu, Yuan Deng, Xiao-Yan Huang, Zhen-Zhen Li, Guo-Qing Fang, Dong Yang, Feng-Liu Wang, Wang Kang, En-Zhi Shen and Chun-Qing Song, was originally published electronically on the publisher’s internet portal on 14 September without open access. With the author(s)’ decision to opt for Open Choice the copyright of the article changed on 12 October to © The Authors 2022 and the article is forthwith distributed under a Creative Commons Attribution 4.0 International License, which permits use, sharing, adaptation, distribution and reproduction in any medium or format, as long as you give appropriate credit to the original author(s) and the source, provide a link to the Creative Commons licence, and indicate if changes were made.

The images or other third party material in this article are included in the article’s Creative Commons licence, unless indicated otherwise in a credit line to the material. If material is not included in the article’s Creative Commons licence and your intended use is not permitted by statutory regulation or exceeds the permitted use, you will need to obtain permission directly from the copyright holder.

To view a copy of this licence, visit http://creativecommons.org/licenses/by/4.0/.

The original article has been corrected.

