# Supplementary material for: CRISPR FISHer enables high-sensitivity imaging of nonrepetitive DNA in living cells through phase separation-mediated signal amplification
Source: Cell Res. 2022 Sep 14;32(11):969–81. doi: 10.1038/s41422-022-00712-z (PMC9652286; doi:10.1038/s41422-022-00712-z)
Supplement: Supplementary file 1 — Fig. S1 [file 41422_2022_712_MOESM1_ESM.pdf]

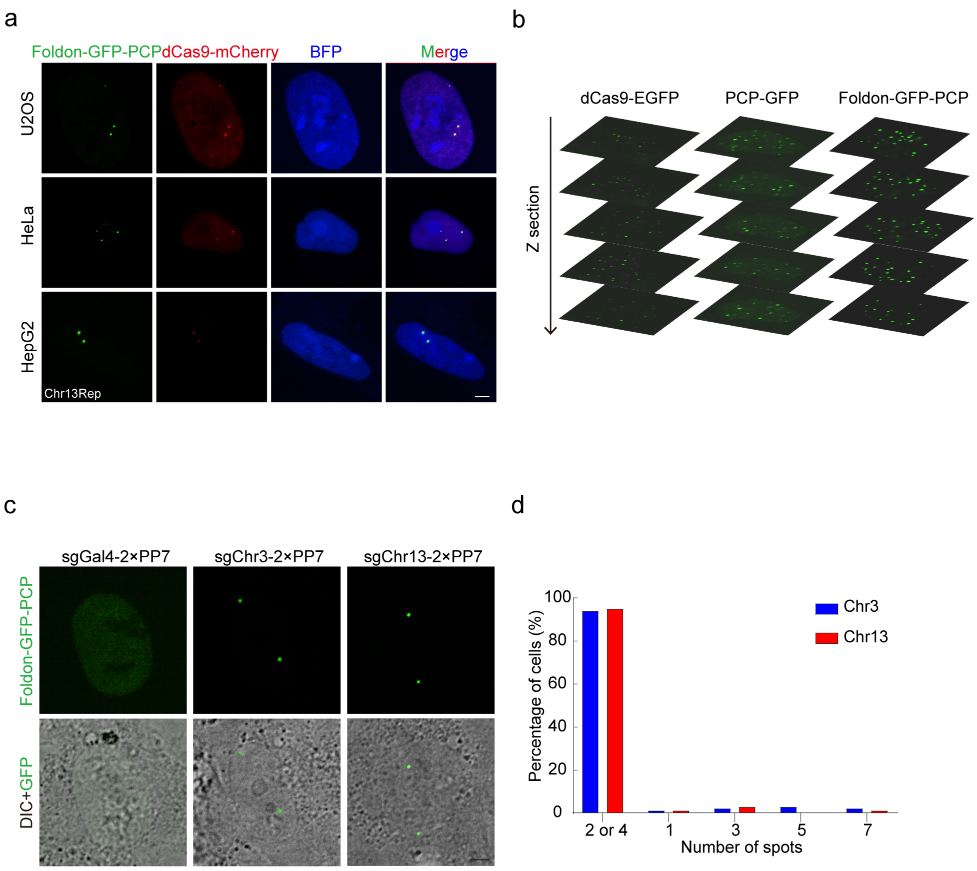

Supplementary Figure 1 Trimeric foldon-GFP-PCP enables robust repetitive genomic loci tracking by CRISPR FISHer in multiple cell lines. (**a**) Dual-color CRISPR imaging showing colocalization of foldon-GFP-PCP (green) and dCas9-mCherry (red) on the Chr13Rep locus in U2OS, HeLa, and HepG2 cells. Scale bar, 5 µm. (**b**) Representative serial Z sections of telomere loci for Fig. 2D. (**c** and **d**) CRISPR FISHer labeled Chr3Rep and Chr13Rep in RPE cells. sgGal4 was used as the control sgRNA. **d** The percentage of cells with different numbers of GFP spots.
