# Supplementary material for: CRISPR FISHer enables high-sensitivity imaging of nonrepetitive DNA in living cells through phase separation-mediated signal amplification
Source: Cell Res. 2022 Sep 14;32(11):969–81. doi: 10.1038/s41422-022-00712-z (PMC9652286; doi:10.1038/s41422-022-00712-z)
Supplement: Supplementary file 2 — Fig. S2 [file 41422_2022_712_MOESM2_ESM.pdf]

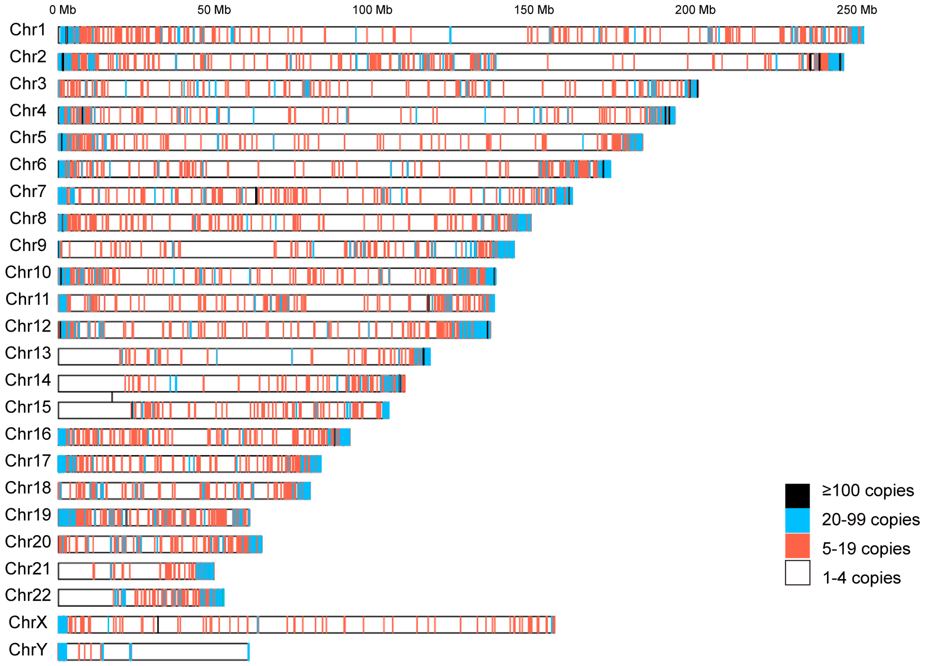

Supplementary Figure 2 The distribution of chromosome-specific repeated elements in the human genome. The raw data were obtained from CRISPRbar (http://genome.ucf.edu/CRISPRbar/).
