# Supplementary material for: CRISPR FISHer enables high-sensitivity imaging of nonrepetitive DNA in living cells through phase separation-mediated signal amplification
Source: Cell Res. 2022 Sep 14;32(11):969–81. doi: 10.1038/s41422-022-00712-z (PMC9652286; doi:10.1038/s41422-022-00712-z)
Supplement: Supplementary file 3 — Fig. S3 [file 41422_2022_712_MOESM3_ESM.pdf]

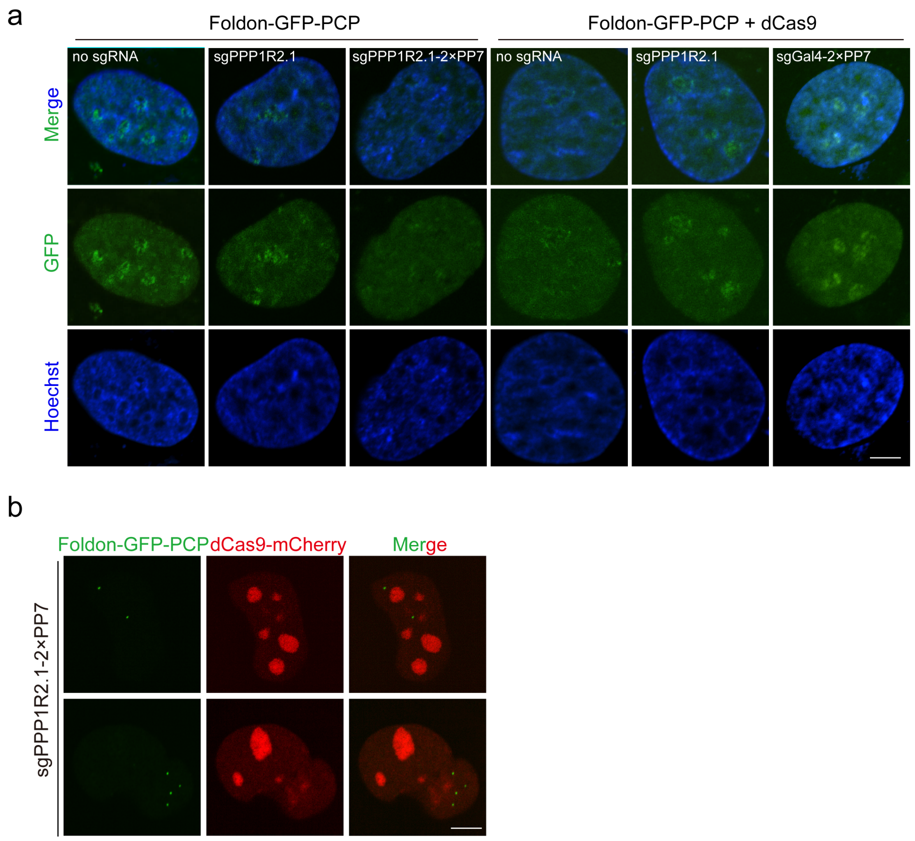


**Supplementary Figure 3 CRISPR FISHer imaging controls in U2OS cells. (a)** CRISPR FISHer imaging controls without dCas9 or with unrelated sgRNAs in U2OS cells. sgPPP1R2.1, regular sgRNA; sgPPP1R2.1-2×PP7 or sgGal4-2×PP7, engineered sgRNA scaffold with PP7 RNA motif. Hoechst 33342 labels the nucleus (blue). Scale bar, 5 µm. (**b**) dCas9-mCherry used in CRISPR FISHer to label nonrepetitive genomic *PPP1R2* locus.
