# Supplementary material for: CRISPR FISHer enables high-sensitivity imaging of nonrepetitive DNA in living cells through phase separation-mediated signal amplification
Source: Cell Res. 2022 Sep 14;32(11):969–81. doi: 10.1038/s41422-022-00712-z (PMC9652286; doi:10.1038/s41422-022-00712-z)
Supplement: Supplementary file 4 — Fig. S4 [file 41422_2022_712_MOESM4_ESM.pdf]

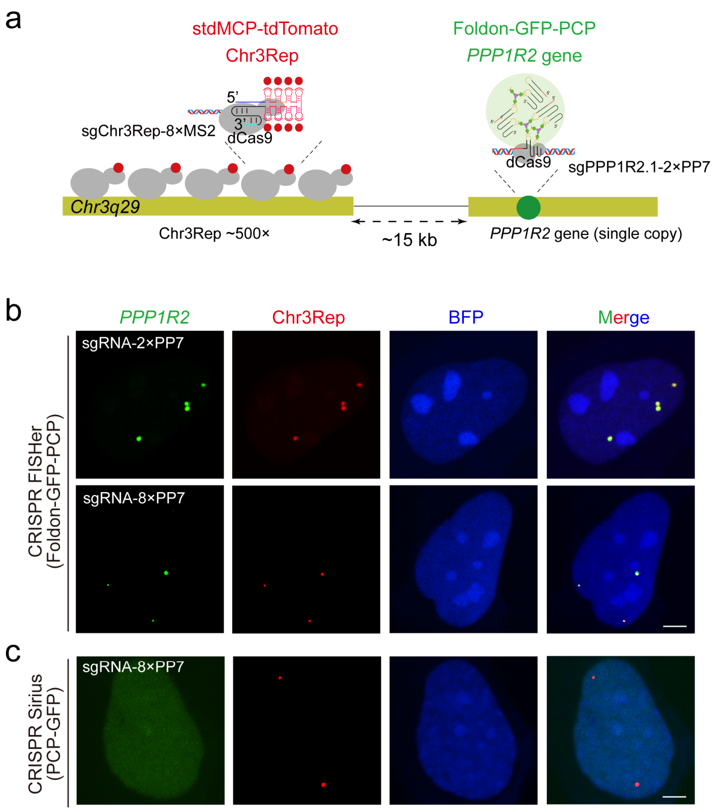

Supplementary Figure 4 CRISPR FISHer visualization of nonrepetitive sequences in the *PPP1R2* gene in live U2OS cells. (**a**) Schematic of dual-color CRISPR imaging shows the loci of *PPP1R2* (GFP) and Chr3Rep (tdTomato) in U2OS cells. (**b** and **c**) Comparison of foldon-GFP-PCP and PCP-GFP labeling of single-copy gene *PPP1R2*. sgPPP1R2.1-2×PP7 or sgPPP1R2.1-8×PP7 was used for targeting the *PPP1R2* gene (green); sgChr3Rep-8×MS2 was used for labeling Chr3Rep loci (red, internal control). BFP: used as an indicator of the nuclei and expression of sgRNA-2×PP7 or sgRNA-8×PP7. Scale bar, 5 µm.
