# Supplementary material for: CRISPR FISHer enables high-sensitivity imaging of nonrepetitive DNA in living cells through phase separation-mediated signal amplification
Source: Cell Res. 2022 Sep 14;32(11):969–81. doi: 10.1038/s41422-022-00712-z (PMC9652286; doi:10.1038/s41422-022-00712-z)
Supplement: Supplementary file 5 — Fig. S5 [file 41422_2022_712_MOESM5_ESM.pdf]

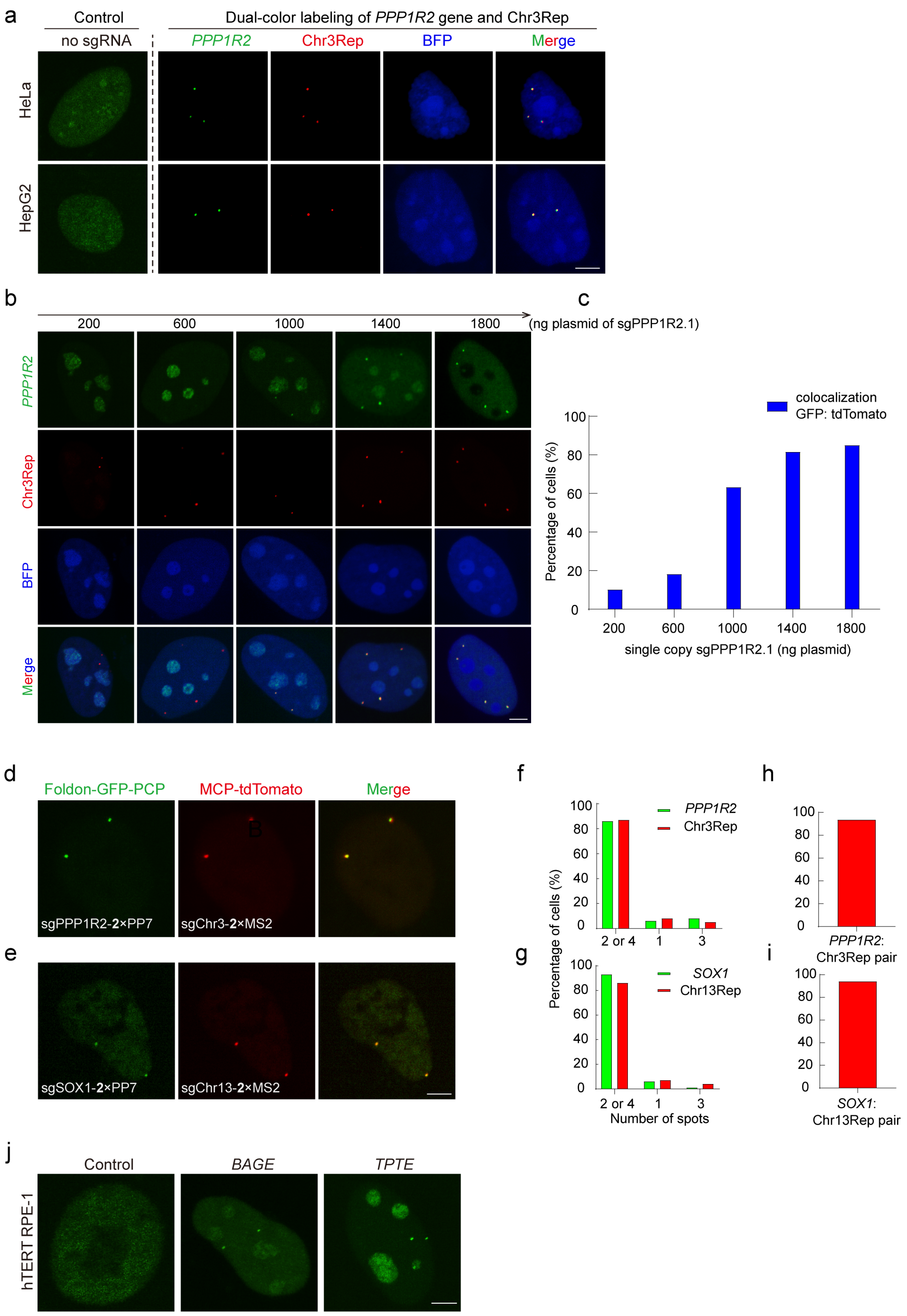

Supplementary Figure 5 CRISPR FISHer imaging of single-copy genes in multiple cell lines. (**a**) Dual-color labeling of PPP1R2 and Chr3Rep in HeLa and HepG2 cells. sgPPP1R2.1-2×PP7 and sgChr3Rep-8×MS2 were used for targeting the *PPP1R2* gene and Chr3Rep, respectively. Scale bar, 5 µm. (**b**) CRISPR FISHer imaging with increasing amount of transfected sgPPP1R2.1 plasmid in U2OS cells. Chr3Rep labeled

by tdTomato was used as internal labeling reference. (**c**) Percentage of cells with co-localization of CRISPR FISHer foci (GFP) and Chr3Rep foci (tdTomato) in (b). (**d** and **e**) Representative CRISPR FISHer imaging for single copy genes *PPP1R2* and *SOX1* in RPE cells. Chr3Rep or Chr13Rep labeled by tdTomato was used as internal labeling reference. (**f)** and (**g**) The number of GFP or tdTomato spots in RPE cells in (d) and (E), respectively. (**h)** and (**i**) Percentage of cells showing co-localization of CRISPR FISHer foci (GFP) and Chr3Rep foci (tdTomato) in (d) and (e), respectively. (**j**) CRISPR FISHer imaging of the single copy genes *BAGE* and *TPTE* located in the heterochromatin regions. Scale bar, 5 μm.
