# Supplementary material for: CRISPR FISHer enables high-sensitivity imaging of nonrepetitive DNA in living cells through phase separation-mediated signal amplification
Source: Cell Res. 2022 Sep 14;32(11):969–81. doi: 10.1038/s41422-022-00712-z (PMC9652286; doi:10.1038/s41422-022-00712-z)
Supplement: Supplementary file 6 — Fig. S6 [file 41422_2022_712_MOESM6_ESM.pdf]

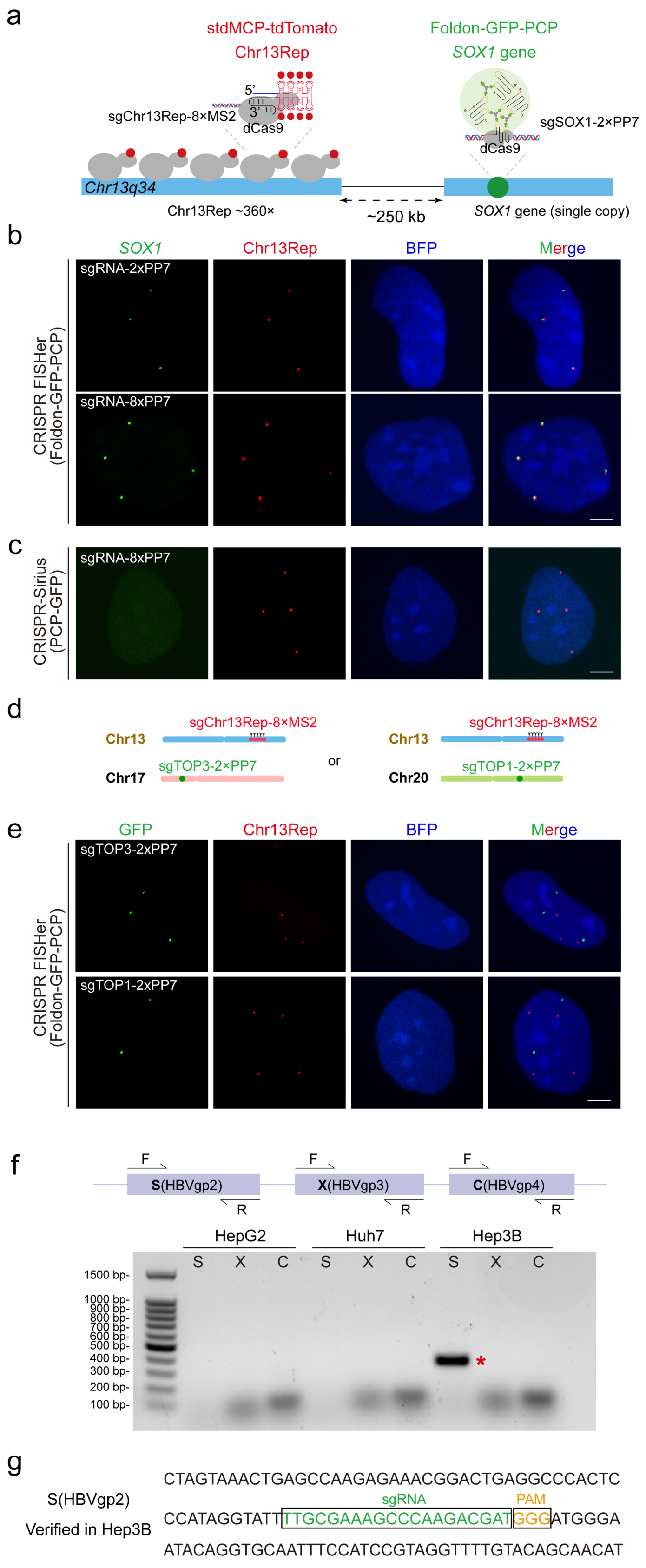
a
stdMCP-tdTomato
Chr13Rep
Foldon-GFP-PCP
SOX1 gene
5'
3'
sgChr13Rep-8×MS2
dCas9
sgSOX1-2×PP7
dCas9
Chr13q34
Chr13Rep ~360×
~250 kb
SOX1 gene (single copy)
b
SOX1
Chr13Rep
BFP
Merge
CRISPR FISHer (Foldon-GFP-PCP)
sgRNA-2xPP7
sgRNA-8xPP7
c
CRISPR-Sirius (PCP-GFP)
sgRNA-8xPP7
d
sgChr13Rep-8×MS2
Chr13
sgTOP3-2×PP7
Chr17
or
sgChr13Rep-8×MS2
Chr13
sgTOP1-2×PP7
Chr20
e
GFP
Chr13Rep
BFP
Merge
CRISPR FISHer (Foldon-GFP-PCP)
sgTOP3-2xPP7
sgTOP1-2xPP7
f
F
S(HBVgp2)
R
F
X(HBVgp3)
R
F
C(HBVgp4)
R
HepG2
Huh7
Hep3B
S X C S X C S X C
1500 bp-
1000 bp-
900 bp-
800 bp-
700 bp-
600 bp-
500 bp-
400 bp-
300 bp-
200 bp-
100 bp-
*
g
S(HBVgp2)
Verified in Hep3B
CTAGTAAACTGAGCCAAGAGAAACGGACTGAGGCCCACTC
sgRNA
PAM
CCATAGGTATTTTGCGAAAGCCCAAGACGATGGGATGGGA
ATACAGGTGCAATTTCCATCCGTAGGTTTTGTACAGCAACAT

Supplementary Figure 6 CRISPR FISHer enables the visualization of nonrepetitive genomic sequences. (**a**) Schematic diagram of CRISPR imaging for loci *SOX1* (GFP) and Chr13Rep (tdTomato). (**b** and **c**) Comparison of foldon-GFP-PCP and PCP-GFP labeling of single-copy gene *SOX1*. sgSOX1-2×PP7 or sgSOX1-8×PP7 was used for targeting the *SOX1* gene (green); sgChr13Rep-8×MS2 was used for labeling Chr13Rep loci (red, internal control). (**d** and **e**) Visualization of single-copy gene *TOP3* or *TOP1* by CRISPR FISHer in U2OS cells. (d) Schematic of target loci on Chr13 and Chr17 or Chr20. (e) Representative images for *TOP3* or *TOP1* gene (green, foldon-GFP-PCP) and Chr13Rep (red, stdMCP-tdTomato, internal control). (**f**) Amplification of S(HBVgp2) by PCR analysis of genomic DNAs of HepG2, Huh7 and Hep3B cells. Red star showing identified HBV band. S, S(HBVgp2); X, X(HBVgp3); C, C(HBVgp4). F, forward primer; R, reverse primer. (**g**) The used sgRNA targeting at S(HBVgp2). Black Box showing sgRNA targeting sequence in Hep3B. Scale bars in (b), (c), and (e), 5 μm.
