# Supplementary material for: CRISPR FISHer enables high-sensitivity imaging of nonrepetitive DNA in living cells through phase separation-mediated signal amplification
Source: Cell Res. 2022 Sep 14;32(11):969–81. doi: 10.1038/s41422-022-00712-z (PMC9652286; doi:10.1038/s41422-022-00712-z)
Supplement: Supplementary file 7 — Fig. S7 [file 41422_2022_712_MOESM7_ESM.pdf]

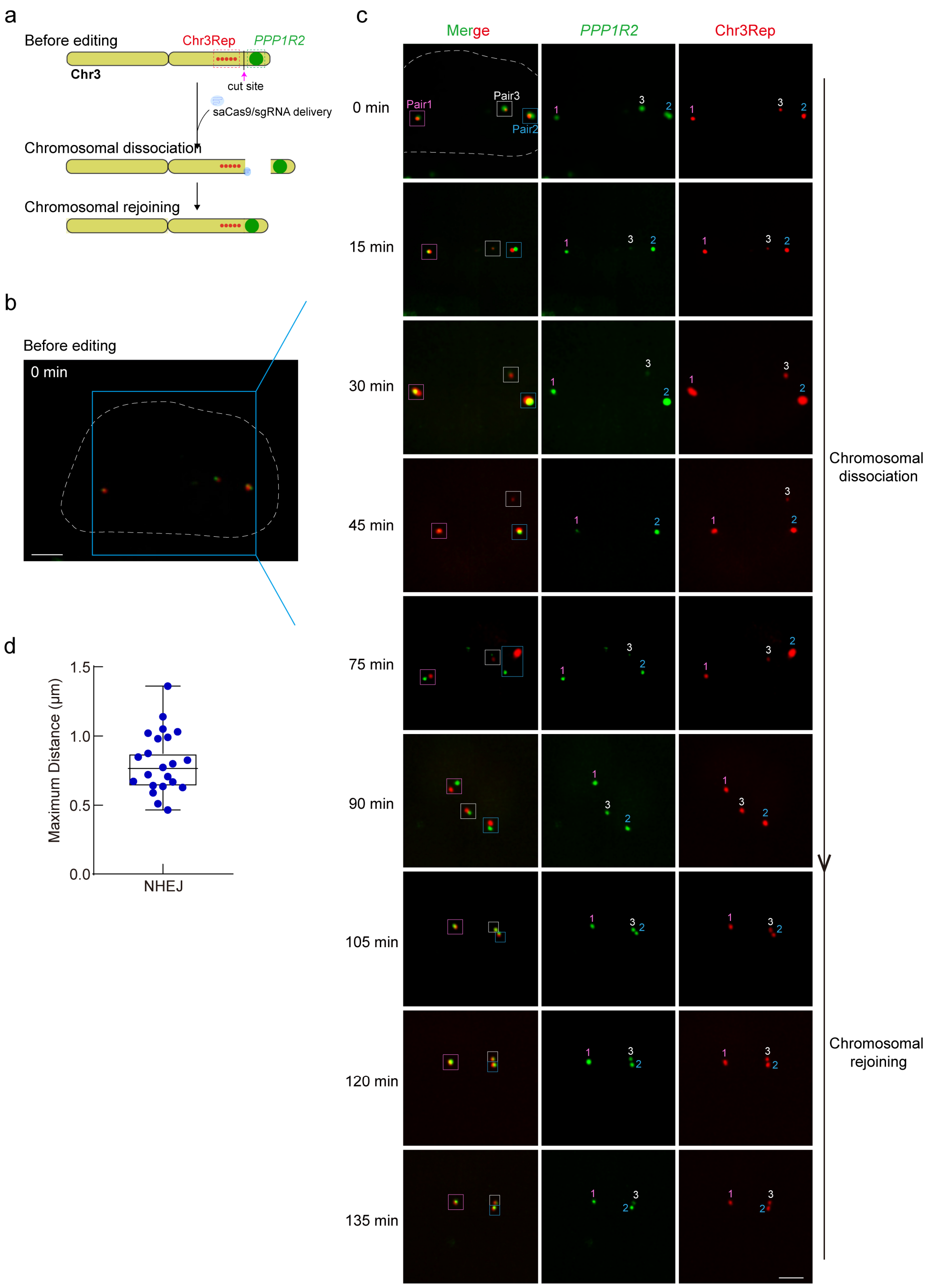

Supplementary Figure 7 Visualization of the dynamics of intrachromosomal dissociation and rejoining through labeling two DSB end fragments after DSB. (**a**) Chr3Rep and *PPP1R2* gene on Chr3 were labeled by CRISPR Sirius and CRISPR FISHer. SaCas9/sgRNA was used to induce DSB between the two labeled loci. (**b** and **c**) The whole U2OS cell time-lapse imaging of DSB-induced chromosomal dissociation

and rejoining. Yellow, blue, and white boxes show DNA loci pairs 1, 2, and 3, respectively. Green, *PPP1R2* gene loci; Red, Chr3Rep loci. Related to Fig. 5a to f. Scale bar, 5 μm. (**d**) Maximum separated distance between the GFP and tdTomato foci during NHEJ in U2OS cells.
