# Supplementary material for: CRISPR FISHer enables high-sensitivity imaging of nonrepetitive DNA in living cells through phase separation-mediated signal amplification
Source: Cell Res. 2022 Sep 14;32(11):969–81. doi: 10.1038/s41422-022-00712-z (PMC9652286; doi:10.1038/s41422-022-00712-z)
Supplement: Supplementary file 8 — Fig. S8 [file 41422_2022_712_MOESM8_ESM.pdf]

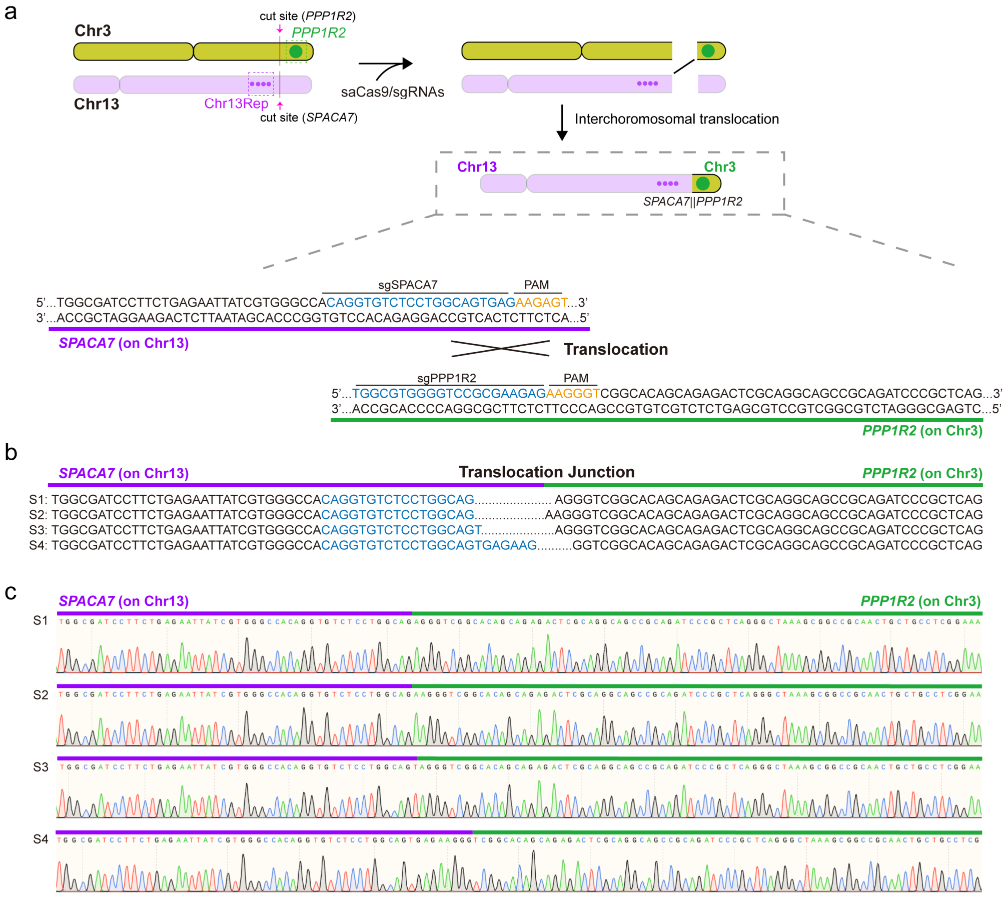

Supplementary Figure 8 Sequencing analysis of interchromosomal translocation between Chr3 and Chr13. Related to Figs. 5g, h. (**a**) Schematic of DSB-induced interchromosomal rejoining between Chr3 and Chr13. Same strategy as in Fig. 4a. Enlarged part showing the proposed translocation between small Chr3 fragment (green, *PPP1R2*) and large Chr13 fragment (purple, Chr13Rep). The gRNA targeting sites for saCas9 are shown in blue and PAMs are shown in orange. (**b** and **c**) The identified sequences of interchromosomal translocation segments for Chr3 (green, small fragment): Chr13 (purple). (b) Four different translocated fragments (S1-S4) were detected in 25 sequenced clones. The verified sequence contains missing nucleotides among translocated junctions. (c) Sanger sequencing maps of interchromosomal

rejoining regions S1-S4 in (b). Sequences mapped to Chr13 were marked with purple color and those mapped to Chr3 were marked green.
