# Supplementary material for: CRISPR FISHer enables high-sensitivity imaging of nonrepetitive DNA in living cells through phase separation-mediated signal amplification
Source: Cell Res. 2022 Sep 14;32(11):969–81. doi: 10.1038/s41422-022-00712-z (PMC9652286; doi:10.1038/s41422-022-00712-z)
Supplement: Supplementary file 9 — Fig. S9 [file 41422_2022_712_MOESM9_ESM.pdf]

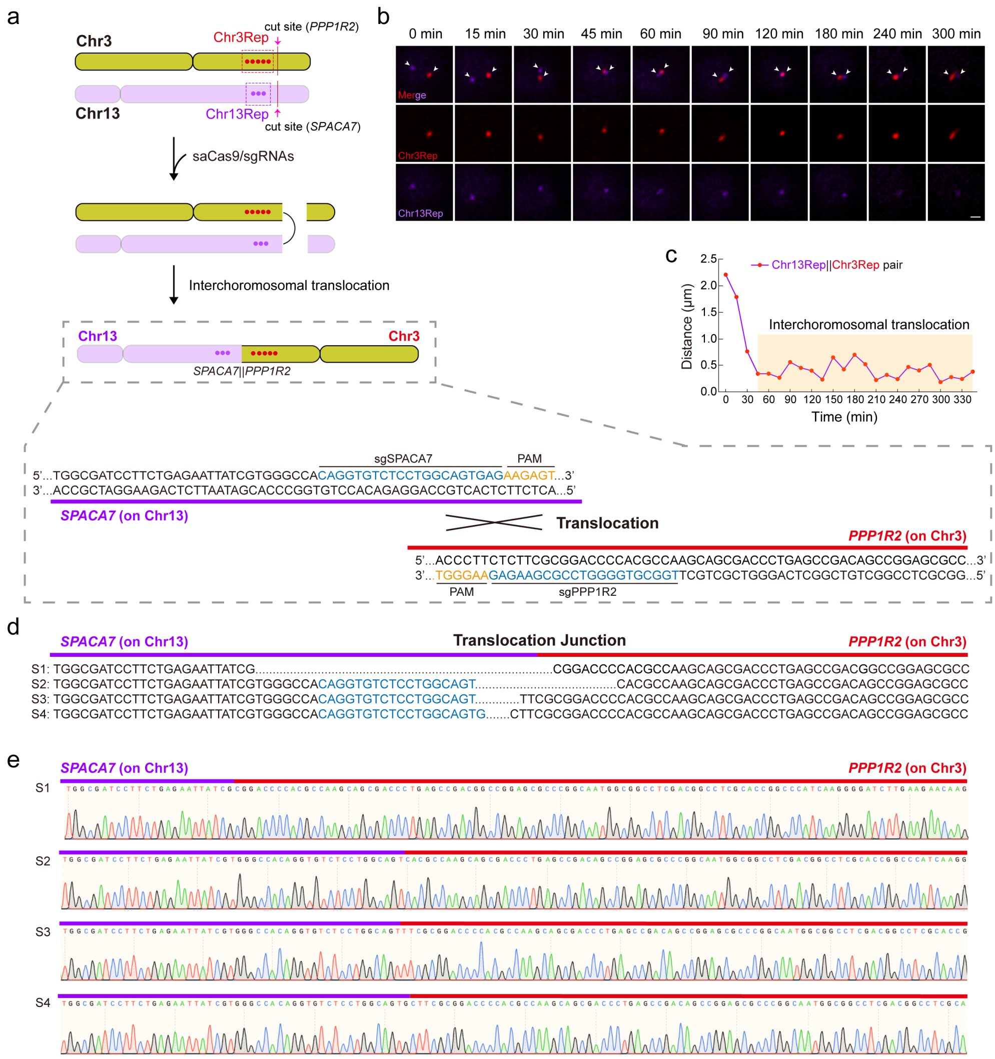

Supplementary Figure 9 Real-time visualization and sequencing verification of DSB-induced interchromosomal translocation between Chr3 and Chr13. (**a**) Schematic of interchromosomal rejoining between Chr3 and Chr13. Same strategy as in Fig. 5g. Enlarged part showing the proposed translocation between large Chr3 fragment (red, Chr3Rep) and large Chr13 fragment (purple, Chr13Rep). The gRNA targeting sites for saCas9 are shown in blue and PAMs are shown in orange. (**b**) Representative images showing chromosomal interchromosomal translocation between Chr3 (red) and Chr13 (purple). (**c**) The distance between a pair of Chr3 (Red) and Chr13 (Purple) loci (arrow) in (b) over time. (**d** and **e**) The identified sequences of

interchromosomal translocation segments for Chr3 (red, large fragment): Chr13 (purple). (d) Four different translocated fragments (S1-S4) were detected in 17 sequenced clones. The verified sequence contains missing nucleotides among translocated junctions. (e) Sanger sequencing maps of interchromosomal rejoining regions S1-S4 in (d). Sequences mapped to Chr13 marked with purple color and those mapped to Chr3 marked red.
