# Supplementary material for: CRISPR FISHer enables high-sensitivity imaging of nonrepetitive DNA in living cells through phase separation-mediated signal amplification
Source: Cell Res. 2022 Sep 14;32(11):969–81. doi: 10.1038/s41422-022-00712-z (PMC9652286; doi:10.1038/s41422-022-00712-z)
Supplement: Supplementary file 10 — Fig. S10 [file 41422_2022_712_MOESM10_ESM.pdf]

a

Chromosome	Start	End	Discordants	Circle score	Mean coverage	Standard deviation	Gene Name
Chr20	49219269	49219302	0	12856	1224.212	20.83376859	RIPOR3
Chr16	23850953	23852505	24	7988.66	559.2442	74.6298186	PRKCB
Chr1	154579986	154580690	2	3455	179.5497	58.35566718	ADAR
Chr19	14720949	14721570	7	2953.29	142.839	13.31822193	CLEC17A
Chr2	175287496	175289218	3	2858.87	153.2091	29.6125654	SCRN3
Chr12	127508271	127508676	0	1790.42	62.06667	11.43928686	LINC02405
Chr6	107429665	107430566	4	1750.98	60.04218	13.20404192	BEND3
ChrX	1522536	1526818	3	1671.95	644.4003	608.726078	ASMTL,ASMTL-AS1
Chr1	7566590	7567174	4	1562	42.06336	9.005007722	CAMTA1
Chr6	89902544	89902979	6	1554	49.23678	10.18389663	GABRR1

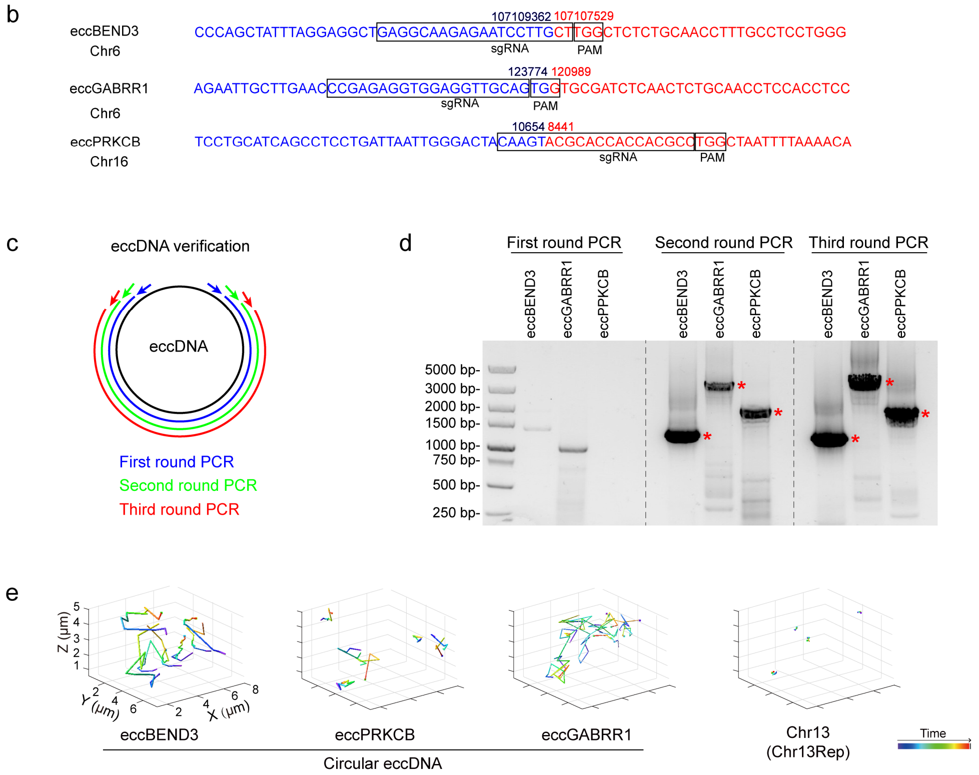

Supplementary Figure 10 Identification, verification, and real-time trajectory tracking of eccDNAs in HepG2 cells. Related to Fig. 6. (**a**) Potential eccDNAs identified in HepG2 cells. (**b**) Junctional sequences of eccDNAs. Boxes indicate sgRNA target sites for each eccDNA. (**c** Strategy for eccDNA verification by three-round PCRs. Primer information is shown in Fig. S12. (**d**) PCR amplification of eccDNA. Red stars indicate the amplified target eccDNA, subjected to purification and sequencing. (**e**) Representative XYZ-t trajectories for circular eccDNAs and Chr13 during a 5-min period. See Movie S6-8 for dynamics.
