# Supplementary material for: CRISPR FISHer enables high-sensitivity imaging of nonrepetitive DNA in living cells through phase separation-mediated signal amplification
Source: Cell Res. 2022 Sep 14;32(11):969–81. doi: 10.1038/s41422-022-00712-z (PMC9652286; doi:10.1038/s41422-022-00712-z)
Supplement: Supplementary file 11 — Fig. S11 [file 41422_2022_712_MOESM11_ESM.pdf]

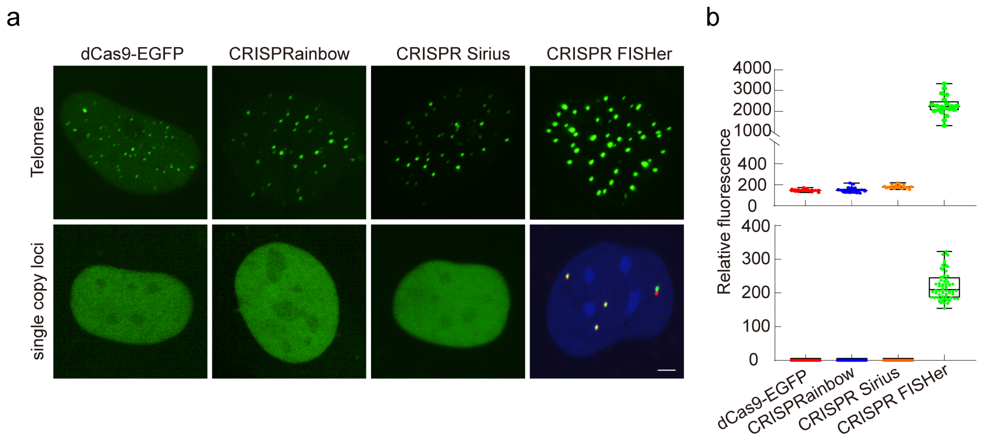

c

	dCas9-EGFP	CRISPRainbow	CRISPR Sirius	CRISPR FISHer
sgRNA scaffold	normal	2PP7/2MS2/2Boxb	8PP7/8MS2	2-8PP7
Protein		PCP-/MCP-/N22-	PCP-/MCP-	Foldon-GFP-PCP
Copy number of target loci for limit resolution	>100 copies	>100 copies	>22 copies	singel copy
Repeat loci imaging	yes	yes	yes	yes
Single copy loci imaging	no	no	no	yes
Signal to bacground	low	low	low	high
Sensitivity	low	low	medium	high
Live cell imaging	yes	yes	yes	yes
Advantages	✓ Live cell imaging<br>✓ Multiple fluorescent protein could be used	✓ Live cell imaging<br>✓ Multiple fluorescent protein could be used<br>✓ Low nucleolus signal	✓ Live cell imaging<br>✓ Multiple fluorescent protein could be used<br>✓ Low nucleolus signal	✓ Live cell imaging<br>✓ Single copy loci imaging<br>✓ High sensitivity<br>✓ High resolution
Disadvantages	✓ High background signal<br>✓ Only for repeat loci imaging<br>✓ High nucleolus signal	✓ High background signal<br>✓ Only for repeat loci imaging	✓ High background signal<br>✓ Only for repeat loci imaging	✓ Label target DNA with GFP

Supplementary Figure 11 Comparison of CRISPR FISHer with other CRISPR based DNA labeling. (**a** and **b**) Representative images and fluorescence data at telomere and single-copy loci. (**c**) Comparison of CRIPSR FISHer with dCas9-EGFP, CRISPRainbow, and CRISPR Sirius.
