# Supplementary material for: CRISPR FISHer enables high-sensitivity imaging of nonrepetitive DNA in living cells through phase separation-mediated signal amplification
Source: Cell Res. 2022 Sep 14;32(11):969–81. doi: 10.1038/s41422-022-00712-z (PMC9652286; doi:10.1038/s41422-022-00712-z)
Supplement: Supplementary file 12 — Fig. S12 [file 41422_2022_712_MOESM12_ESM.pdf]

Supplementary Figure 12. Primers used in this study

HBV S gene (Fig. S6)

S(HBVgp2)-F	CTAATTCCAGGATCCTCAACAACCAGCACG
S(HBVgp2)-R	GAAAATTGGTAACAGCGGTAAAAAGGGACT
X(HBVgp3)-F	CGCTTGTTTTGCTCGCAGCAGGTCTGGAGCAA
X(HBVgp3)-R	CAGTCTTTAAACAAACAGTCTTTGAAGTAT
C(HBVGP4)-F	TGGCTTTGGGGCATGGACATCGACCCTTAT
C(HBVGP4)-R	TCATTAACTGTGAGTGGGCCTACAAACTGT

Translocation (Fig. S8)

GFP/Halo-F	ATTGAGTTTCCAAAACATATTTTCA
GFP/Halo-R	CCGCGAGCCGCGGGTCAAGTGCCAG

Translocation (Fig. S9)

tdTomato/Halo-F	GTCAGGAATTATCTCCAGGGTC
tdTomato/Halo-R	GGAAGCATGATAAACGTCATCG

eccDNA PCR (Fig. S11)

eccBEND3:

1st round PCR-F	CACTAGTCAGAAATAACTGAAAATCAGACA
1st round PCR-R	TCAGTATCAGGGCATTGATTGCTCAATTGA
2nd round PCR-F	ATTAAGAGGGTTGATTGATTGATTGATTTG
2nd round PCR-R	ATAAGTCATTTACTGGCCTTTTCTTGTATG
3rd round PCR-F	AGATGGAGTCTCGCTCTGTTGCCCAGGATG
3rd round PCR-R	TCAGGGGCTGTTTTGGTTTGAGCATGGAGC

eccGABRR1:

1st round PCR-F	ACCCACTGCACTGCTGCATTCCTCATTTGT
1st round PCR-R	TGAGAACAGTTTCCTAGGCACCAGCATACA
2nd round PCR-F	GTCCTAAGCATTGGGTTCTGTGCCTGTCTC
2nd round PCR-R	ACTGAAATCCACAAGTGAGATACTCTATTA
3rd round PCR-F	CCACTAGCCTGCAGTCCTAGGAAGGACAGT
3rd round PCR-R	GCTTGGTAGATTTTACTAAGGTAATATTGA

eccPRKCB:

1st round PCR-F	AAGATCCTTCTACCTACATCCTCTTAGCTA
1st round PCR-R	TTGTCACTCCCCAGGGATAGTGGAGCAGAA
2nd round PCR-F	TAATCACATACCCACACCTTGCTGTGAGGG
2nd round PCR-R	ACTCAAAGGAACCTGAGGCCCTGGGCTC
3rd round PCR-F	GCATGTGGGAAATGTAGTGTTTATTTCAGG
3rd round PCR-R	GGATTGCTGACTTAAGTGAGATCATAAAAC
