# Supplementary material for: CRISPR FISHer enables high-sensitivity imaging of nonrepetitive DNA in living cells through phase separation-mediated signal amplification
Source: Cell Res. 2022 Sep 14;32(11):969–81. doi: 10.1038/s41422-022-00712-z (PMC9652286; doi:10.1038/s41422-022-00712-z)
Supplement: Supplementary file 13 — Fig. S13 [file 41422_2022_712_MOESM13_ESM.pdf]

Supplementary Figure 13. sgRNAs used in this study.

SpCas9 sgRNAs:

sgPPP1R2.1	GAGGACTGGCTAAGCTTAGA
sgPPP1R2.2	TCTATTCTAGAATACGTAGT
sgSOX1	GCTACGCGCACGTCAACGGCT
sgTOP3	GCAGACCCCCGATCCACGCT
sgTOP1	CGCGGCAACCACCCCAAGAT
sgTPTE	TGATTCTAACGACCCTATTA
sgBAGE	GAGCAATAGGAGGGTAACCG
sgeccBEND3	GAGGCAAGAGAATCCTTGCT
sgeccPRKCB	CAAGTACGCACCACCACGCC
sgeccGABRR1	CCGAGAGGTGGAGGTTGCAG
sgChr3Rep	TGATATCACAG
sgChr13Rep	ACCATTCCTTC
sgTelomere	TTAGGGTTAGGGTTAGGGTT
sgTBG	ACCATCCCAGGGTTAATGCT
sgHBV	TTGCGAAAGCCCAAGACGAT

SaCas9 sgRNAs:

sgchr3	TGGCGTGGGGTCCGCGAAGAG
sgchr13	CAGGTGTCTCCTGGCAGTGAG

SpCas9 sgRNAs used in RNP:

sgChr3	GCTCCTCGTCGACATTCCCG

sgChr13	AATTCTCAGAAGGATCGCCA
