# Supplementary material for: CRISPR FISHer enables high-sensitivity imaging of nonrepetitive DNA in living cells through phase separation-mediated signal amplification
Source: Cell Res. 2022 Sep 14;32(11):969–81. doi: 10.1038/s41422-022-00712-z (PMC9652286; doi:10.1038/s41422-022-00712-z)
Supplement: Supplementary file 14 — Fig. S14 [file 41422_2022_712_MOESM14_ESM.pdf]

Supplementary Figure 14. Sequences of RNA coating proteins used in this study.

Foldon-linker-GFP-PCP

MGYIPEAPRDGQAYVRKDGEWVLLSTFLSGGGGSGGGGSGGGGSRKGEELF
TGVVPILVELDGDVNGHKFSVRGEGEGDATNGKLTLKFICTTGKLPVPWPTL
VTTLTYGVQCFARYPDHMKQHDFFKSAMPEGYVQERTISFKDDGTYKTRAE
VKFEGDTLVNRIELKGIDFKEDGNILGHKLEYNFNSHNVYITADKQKNGIKAN
FKIRHNVEDGSVQLADHYQQNTPIGDGPVLLPDNHYLSTQSVLSKDPNEKRD
HMVLLEFVTAAGITHGMDELYKMGSKTIVLSVGEATRTLTEIQSTADRQIFEE
KVGPLVGRLRLTASLRQNGAKTAYRVNLKLDQADVVDSGLPKVRYTQVWS
HDVTIVANSTEASRKSLYDLTKSLVATSQVEDLVVNLVPLGRGGGGTSGGGS
GS

stdMCP-tdtomato

ASNFTQFVLVDNGGTGDVTVAPSNFANGIAEWISSNSRSQAYKVTCSVRQSS
AQNRKYTIKVEVPKGAWRSYLNMELTIPIFATNSDCELIVKAMQGLLKDGNPI
PSAIAANSGIYAMASNFTQFVLVDNGGTGDVTVAPSNFANGIAEWISSNSRSQ
AYKVTCSVRQSSAQNRKYTIKVEVPKGAWRSYLNMELTIPIFATNSDCELIVK
AMQGLLKDGNPIPSAIAANSDSRMVSKGEEVIKEFMRFKVRMEGSMNGHEFE
IEGEGEGRPYEGTQTAKLKVTKGGPLPFAWDILSPQFMYGSKAYVKHPADIP
DYKKLSFPEGFKWERVMNFEDGGLVTVTQDSSLQDGTLIYKVKMRGTNFPP
DGPVMQKKTMGWEASTERLYPRDGVLKGEIHQALKLKDGGHYLVEFKTIY
MAKKPVQLPGYYYVDTKLDITSHNEDYTIVEQYERSEGRHHLFLGHGTGSTG
SGSSGTASSEDNNMAVIKEFMRFKVRMEGSMNGHEFEIEGEGEGRPYEGTQT
AKLKVTKGGPLPFAWDILSPQFMYGSKAYVKHPADIPDYKKLSFPEGFKWER
VMNFEDGGLVTVTQDSSLQDGTLIYKVKMRGTNFPPDGPVMQKKTMGWEA
STERLYPRDGVLKGEIHQALKLKDGGHYLVEFKTIYMAKKPVQLPGYYYVD
TKLDITSHNEDYTIVEQYERSEGRHHLFLYGMDELYK

N22-Halo

MGNARTRRRERRAEKQAQWKAANGGGGTSGSGSAEIGTGFPFDPHYVEVLG
ERMHYVDVGPRDGTPVLFLHGNPTSSYVWRNIIPHVAPTHRCIAPDLIGMGKS
DKPDLGYFFDDHVRFMDAFIEALGLEEVVLVIHDWGSALGFHWAKRNPERV
KGIAFMEFIRPIPTWDEWPEFARETFQAFRTTDVGRKLIIDQNVFIEGTLPMGV
VRPLTEVEMDHYREPFLNPVDREPLWRFPNELPIAGEPANIVALVEEYMDWL
HQSPVPKLLFWGTPGVLIPPAEAARLAKSLPNCKAVDIGPGLNLLQEDNPDLI
GSEIARWLSTLEISG
