# Supplementary material for: CRISPR FISHer enables high-sensitivity imaging of nonrepetitive DNA in living cells through phase separation-mediated signal amplification
Source: Cell Res. 2022 Sep 14;32(11):969–81. doi: 10.1038/s41422-022-00712-z (PMC9652286; doi:10.1038/s41422-022-00712-z)
Supplement: Supplementary file 27 — Supplementary video legends [file 41422_2022_712_MOESM27_ESM.docx]

**Supplementary information, Video S1. Comparison of foldon-GFP-PCP, PCP-GFP, and dCas9-EGFP labeling of telomere loci in U2OS cells.**

Telomeres are labeled by foldon-GFP-PCP, PCP-GFP and dCas9-EGFP respectively. Images were taken with Z confocal planes spaced by 0.2 μm.

**Supplementary information, Video S2. Three-color CRISPR imaging of loci for *PPP1R2* gene (green), Chr3Rep (red), and Chr13Rep (purple) in U2OS cells.**

*PPP1R2* gene is labeled by CRISPR-FISHer. Chr3Rep is labeled by stdMCP-tdTomato. Chr13Rep is labeled by N22-Halo. Images were taken with Z confocal planes spaced by 0.2 μm. Movie shows the 3D rotation of this cell.

**Supplementary information, Video S3.** **CRISPR FISHer tracks the dynamics of eccBEND3 in a representative U2OS cell during 5-min period.**

eccBEND3 is labeled by CRISPR-FISHer. Images were taken with Z confocal planes spaced by 0.2 μm. The trajectory diagram on the right corresponds to the movement of the representative cell fluorescent spots on the left.

**Supplementary information, Video S4.** **CRISPR-Sirius tracks the dynamics of Chr3Rep in a representative U2OS cell during 5-min period.**

Chr3Rep is labeled by CRISPR-Sirius. Images were taken with Z confocal planes spaced by 0.2 μm. The trajectory diagram on the right corresponds to the movement of the representative cell fluorescent spots on the left.

**Supplementary information, Video S5. CRISPR FISHer tracks the dynamics of *PPP1R2* gene in a representative U2OS cell during 5-min period.**

*PPP1R2* gene is labeled by CRISPR-FISHer. Images were taken with Z confocal planes spaced by 0.2 μm. The trajectory diagram on the right corresponds to the movement of the representative cell fluorescent spots on the left.

**Supplementary information, Video S6.** **CRISPR-Sirius tracks the dynamics of Chr13Rep in a representative U2OS cell during 5-min period.**

Chr13Rep is labeled by CRISPR-Sirius. Images were taken with Z confocal planes spaced by 0.2 μm. The trajectory diagram on the right corresponds to the movement of the representative cell fluorescent spots on the left.

**Supplementary information, Video S7.** **CRISPR FISHer tracks the dynamics of eccGABRR1 in a representative U2OS cell during 5-min period.**

eccGABRR1 is labeled by CRISPR-FISHer. Images were taken with Z confocal planes spaced by 0.2 μm. The trajectory diagram on the right corresponds to the movement of the representative cell fluorescent spots on the left.

**Supplementary information, Video S8. CRISPR FISHer tracks dynamics of eccPRKCB in a representative U2OS cell during 5-min period.**

eccPRKCB is labeled by CRISPR-FISHer. Images were taken with Z confocal planes spaced by 0.2 μm. The trajectory diagram on the right corresponds to the movement of the representative cell fluorescent spots on the left.

**Supplementary information, Video S9. CRISPR FISHer tracks dynamics of linear eccBEND3 in a representative normal U2OS cell during 5-min period.**

Linear eccBEND3 is labeled by CRISPR-FISHer. Images were taken with Z confocal planes spaced by 0.2 μm. The trajectory diagram on the right corresponds to the movement of the representative cell fluorescent spots on the left.

**Supplementary information, Video S10.** **CRISPR FISHer tracks dynamics of linear eccPRKCB in a representative normal U2OS cell during 5-min period.**

Linear eccPRKCB is labeled by CRISPR-FISHer. Images were taken with Z confocal planes spaced by 0.2 μm. The trajectory diagram on the right corresponds to the movement of the representative cell fluorescent spots on the left.

**Supplementary information, Video S11.** **CRISPR FISHer tracks dynamics of linear eccGABRR1 in a representative normal U2OS cell during 5-min period.**

Linear eccGABRR1 is labeled by CRISPR-FISHer. Images were taken with Z confocal planes spaced by 0.2 μm. The trajectory diagram on the right corresponds to the movement of the representative cell fluorescent spots on the left.

**Supplementary information, Video S12. CRISPR FISHer tracks dynamics of AAV-TBG in a representative normal U2OS cell during 5-min period.**

AAV is labeled by CRISPR-FISHer. Images were taken with Z confocal planes spaced by 0.2 μm. The trajectory diagram on the right corresponds to the movement of the representative cell fluorescent spots on the left.
